# Distinct hepatic immunological patterns are associated with the progression or inhibition of hepatocellular carcinoma

**DOI:** 10.1016/j.celrep.2022.110454

**Published:** 2022-03-01

**Authors:** Faridoddin Mirshahi, Hussein F. Aqbi, Madison Isbell, Saeed H. Manjili, Chunqing Guo, Mulugeta Saneshaw, Dipankar Bandyopadhyay, Mikhail Dozmorov, Archit Khosla, Katy Wack, Oscar M. Carrasco-Zevallos, Michael O. Idowu, Xiang-Yang Wang, Arun J. Sanyal, Masoud H. Manjili

**Affiliations:** 1) Department of Internal Medicine, VCU School of Medicine, Richmond, VA 23298, USA.; 2) Department of Internal Medicine, VCU School of Medicine, Richmond, VA 23298, USA; VCU Massey Cancer Center, 401 College Street, Richmond, VA 23298, USA; College of Science, Mustansiriyah University, Baghdad, Iraq.; 3) Department of Microbiology & Immunology, VCU School of Medicine, Richmond, VA 23298, USA.; 4) Department of Human & Molecular Genetics, VCU School of Medicine, Richmond, VA 23298, USA.; 5) VCU Massey Cancer Center, 401 College Street, Richmond, VA 23298, USA.; 6) VCU Massey Cancer Center, 401 College Street, Richmond, VA 23298, USA; Department of Biostatistics, Virginia Commonwealth University, Richmond, VA 23298, USA.; 7) PathAI, Inc., Boston, MA 02215, USA.; 8) VCU Massey Cancer Center, 401 College Street, Richmond, VA 23298, USA; Department of Pathology, VCU School of Medicine, Richmond, VA 23298, USA.; 9) VCU Massey Cancer Center, 401 College Street, Richmond, VA 23298, USA; Department of Human & Molecular Genetics, VCU School of Medicine, Richmond, VA 23298, USA; Hunter Holmes McGuire VA Medical Center, Richmond, VA 23298, USA.; 10) Department of Internal Medicine, VCU School of Medicine, Richmond, VA 23298, USA; VCU Massey Cancer Center, 401 College Street, Richmond, VA 23298, USA.; 11) Department of Microbiology & Immunology, VCU School of Medicine, Richmond, VA 23298, USA; VCU Massey Cancer Center, 401 College Street, Richmond, VA 23298, USA; Department of Pathology, VCU School of Medicine, Richmond, VA 23298, USA.

## Abstract

To discover distinct immune responses promoting or inhibiting hepatocellular carcinoma (HCC), we perform a three-dimensional analysis of the immune cells, correlating immune cell types, interactions, and changes over time in an animal model displaying gender disparity in nonalcoholic fatty liver disease (NAFLD)-associated HCC. In response to a Western diet (WD), animals mount acute and chronic patterns of inflammatory cytokines, respectively. Tumor progression in males and females is associated with a predominant CD8^+ >^ CD4^+^, Th1 > Th17 > Th2, NKT > NK, M1 > M2 pattern in the liver. A complete rescue of females from HCC is associated with an equilibrium Th1 = Th17 = Th2, NKT = NK, M1 = M2 pattern, while a partial rescue of males from HCC is associated with an equilibrium CD8^+^ = CD4^+^, NKT = NK and a semi-equilibrium Th1 = Th17 > Th2 but a sustained M1 > M2 pattern in the liver. Our data suggest that immunological pattern-recognition can explain immunobiology of HCC and guide immune modulatory interventions for the treatment of HCC in a gender-specific manner.

## In brief

Mirshahi et al. performed a three-dimensional analysis of hepatic and splenic immune cells, correlating the immune cell types, their interactions and proportions, and changes over time. They discover gender-associated immunological patterns determining tumor progression, as well as partial or complete inhibition of hepatocellular carcinoma.

## Graphical abstract

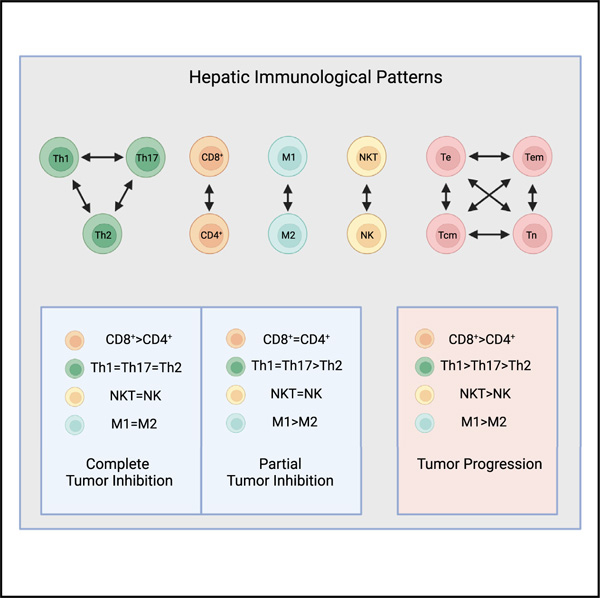

## INTRODUCTION

Over 90% of hepatocellular carcinomas (HCCs) occur in the setting of chronic liver disease, with nonalcoholic steatohepatitis (NASH) being the fastest rising etiology for HCC (Perumpail et al., 2017). In fact, the decrease in virally induced HCC might be offset by increased steatogenic HCC (Baffy, 2013). Because few patients undergo invasive biopsy for diagnosis of NASH, the true prevalence of NASH-associated HCC is underestimated. Nonalcoholic fatty liver disease (NAFLD) and HCC are more prevalent in men than in women (Lonardo et al., 2019; Natarajan et al., 2020). On the other hand, women are four times more likely to have autoimmune hepatitis than men (Manns et al., 2010; Sohn, 2021). This disparity in the prevalence of liver diseases neither is fully understood nor is considered in clinical practice guidelines for patients with NASH or HCC. Although sex hormones could impact the incidence of NAFLD, they are not considered to be major factors, as both premenopausal and postmenopausal women show lower incidence of NAFLD compared with men (Hamaguchi et al., 2012). It appears that gender is a modifier rather than a driving factor for HCC.

Increasing evidence suggests that the immune system plays paradoxical roles in the promotion and inhibition of the hepatic inflammation and HCC. For instance, in a mouse model of chronic liver disease, the adaptive immune system in the liver improved survival of animals during liver damage and paradoxically promoted HCC (Endig et al., 2016). In addition, CD8^+^ T cells and anti-PD1 immunotherapy increased NASH-associated HCC, but the outcome was reversed when tumor necrosis factor alpha (TNF-a) was blocked (Pfister et al., 2021). In the clinic, anti-PD-L1 immunotherapy was less effective against non-viral HCC, while overall survival was similarly improved for both non-viral and viral HCC (Bonilla et al., 2020). In regards to CD4^+^ T cells, while some reports suggest that Th17 cells play no role in liver injury and inhibit HCC (Xie et al., 2010; Zenewicz et al., 2007), other reports show that Th17 cells promote liver injury and HCC (Ma et al., 2020; Nagata et al., 2008). Similarly, M1 macrophages are reported to inhibit HCC and promote liver damage and NASH (Rodríguez et al., 2021; Zhang et al., 2019). This immunological paradox is in large part due to a reductionistic approach by focusing on specific immune cell function or a ‘‘cause-effect’’ direction. A recent review of literature explored the complexity of tumor microenvironment (Fares et al., 2020), suggesting that understanding and modulating the pattern of interactions might be more important than each cellular or molecular component. Discovery of such immunological patterns is feasible by taking a higher dimensional approach correlating immune cell types, their interactions, and functional changes over time. To this end, we took a perturbation three-dimensional approach (Paus, 2005) and demonstrated that the pattern of the immune response reflecting the ratio and proportion of CD4^+^/CD8^+^ T cells, Th1/Th2/Th17 cells, NKT/NK cells, M1/M2 macrophages, and T cell phenotypes as well as an acute versus chronic pattern of inflammation are better indicators for understanding how NAFLD-associated HCC is promoted or inhibited. We found that a predominant hepatic Th1, but not Th17, inflammatory pattern was associated with the promotion of HCC. Also, we demonstrated that an equilibrium Th1 = Th17 = Th2, NKT = NK, M1 = M2 pattern, or predominant Th1 > Th17 > Th2, NKT > NK, M1 > M2 pattern could exhibit opposing outcomes, perhaps because of distinctively collective function independent from the cellular components. Accordingly, therapeutic attempts should focus on discovering methods for immune modulation toward restoration of healthy immunological patterns rather than the induction or suppression of specific immune cell types.

## RESULTS

### Reversal of Western diet to chow diet during NAFLD results in the rescue of DIAMOND mice from steatohepatitis and HCC, with females being more responsive than males

**Figure 1.**
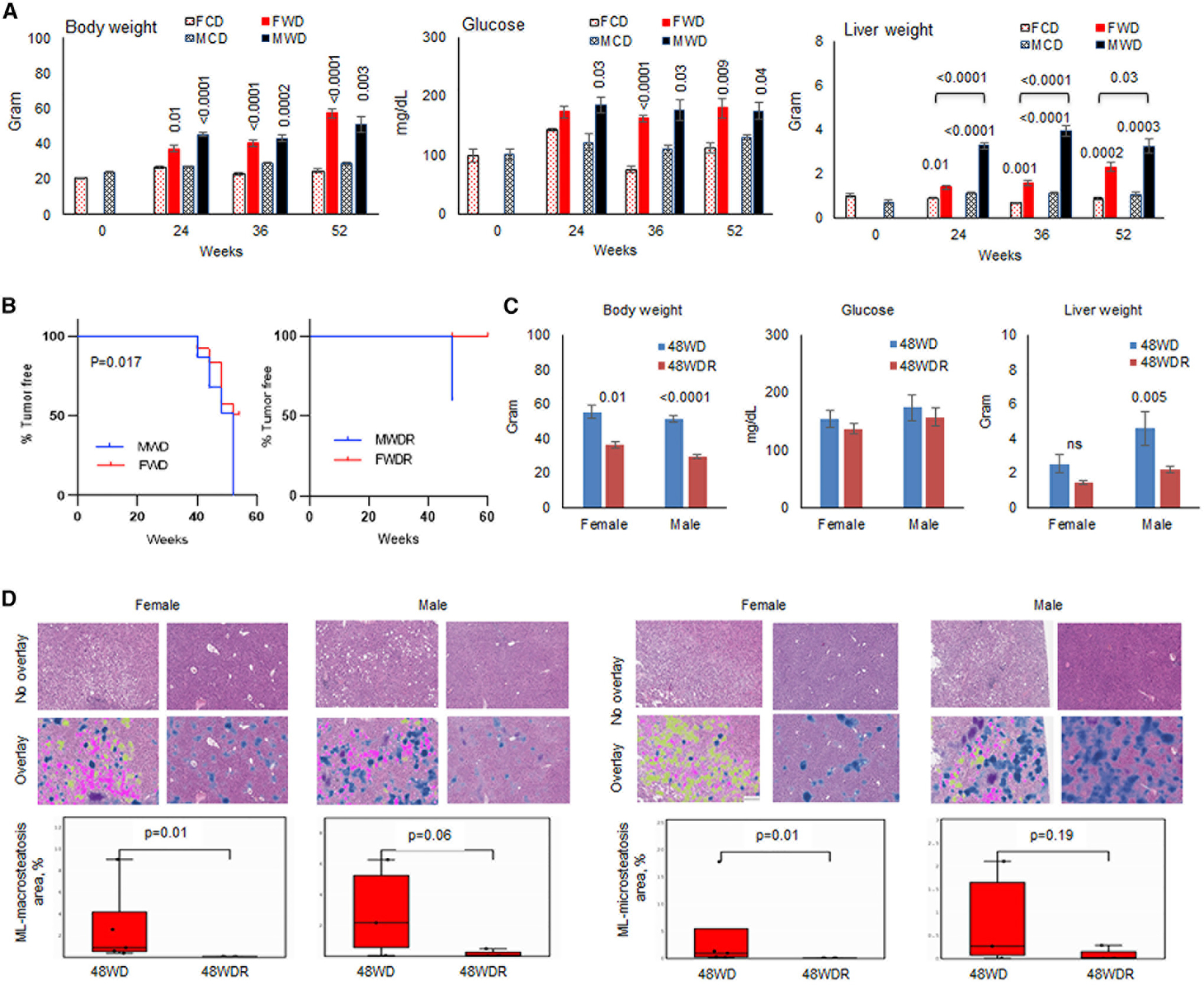
The progression and inhibition of NAFLD to HCC in DIAMOND mice (A) Male or female DIAMOND mice were fed CD (MCD or FCD, 3–10 mice/group) or WD (MWD or FWD, seven mice/group) starting at 10 weeks of age. Mice were sacrificed before starting the diet (week 0) or after 24, 36, and 52 weeks of being on diet. Total body weight, blood glucose, and liver weight were measured. (B) Hepatic tumor incidence in male and female DIAMOND mice after 52 weeks of being on a WD (23 MWD and 25 FWD) or those that their diet was reversed to a CD after 36 weeks of being on a WD and remained on a CD through weeks 48–60 (MWDR and FWDR). (C) Body weight, serum glucose, and liver weight of DIAMOND mice after 48 weeks of being on a WD (48WD, four to six mice/group) or those that their diet was corrected to a CD after 36 weeks of being on a WD and stayed on a CD for additional 12 weeks (48WDR, four to five mice/group). (D) Livers were collected after 48 weeks of being on diet, and H&E staining was performed on formalin fixed paraffin embedded (FFPE) tissues. Machine learning (ML) macrosteatosis and microsteatosis were enumerated using a machine learning model trained to detect and quantitate steatotic cells on whole slide histology images. Macrosteatosis, microsteatosis, and lobular inflammation are shown as pink, yellow, and blue, respectively. Error bars are SEM.

We have previously reported that DIAMOND mice develop NAFLD and NASH, leading to HCC during Western diet (WD) (Asgharpour et al., 2016). In order to determine whether a higher susceptibility of males to HCC was because of their distinct ability to handle fat deposition, biological variables, including blood glucose, body weight, liver weight, and structural fat deposition in the liver and abdominal area, were investigated longitudinally during a WD. Both males and females showed similar patterns of weight gain (Figure 1A, left panel) and elevated blood glucose during WD compared with mice being on a chow diet (CD) (Figure 1A, middle panel). However, liver weight was significantly increased in males compared with females while being on a WD (Figure 1A, right panel). A greater increase in the liver weight of males 24 weeks after being on a WD (during NAFLD) was associated with predominant fat deposition in the liver shown by a fatty discoloration (Figure S2A) compared with females showing a predominant visceral fat deposition as abdominal obesity (Figure S2B). Males and females showed similar patterns of abdominal fat during NASH and HCC (data not shown). Similar observations were made in a mouse model of alcoholic fatty liver disease (AFLD) (Figure S3C).

We wanted to determine whether diet correction during NAFLD and NASH could rescue females and males from HCC. As expected, males showed a significantly higher incidence of HCC with 100% of males and 36% of females developing tumor (Figure 1B, left panel). Reversal of a WD to a CD at week 36 of being on a WD resulted in 60% inhibition of tumor development in males and complete prevention of HCC in females (Figure 1B, right panel). Also, reversal of a WD resulted in a weight loss, but it did not correct high levels of blood glucose in males or females (Figure 1C, left and middle panels). Only males showed a significantly decreased liver weight following a reverse diet (Figure 1C, right panel; p = 0.005). Correction of a WD for females resulted in significant decreases in both macrosteatosis and microsteatosis (p = 0.01) while males did not decrease microsteatosis, with only a marginal decrease in macrosteatosis (p = 0.06; Figure 1D).

### Male and female mice exhibit distinct systemic inflammatory patterns, with males dominating the inflammatory Th17 > Th1 > Th2 pattern and females modulating the Th1 = Th17 > Th2 pattern as they age on a CD

**Figure 2.**
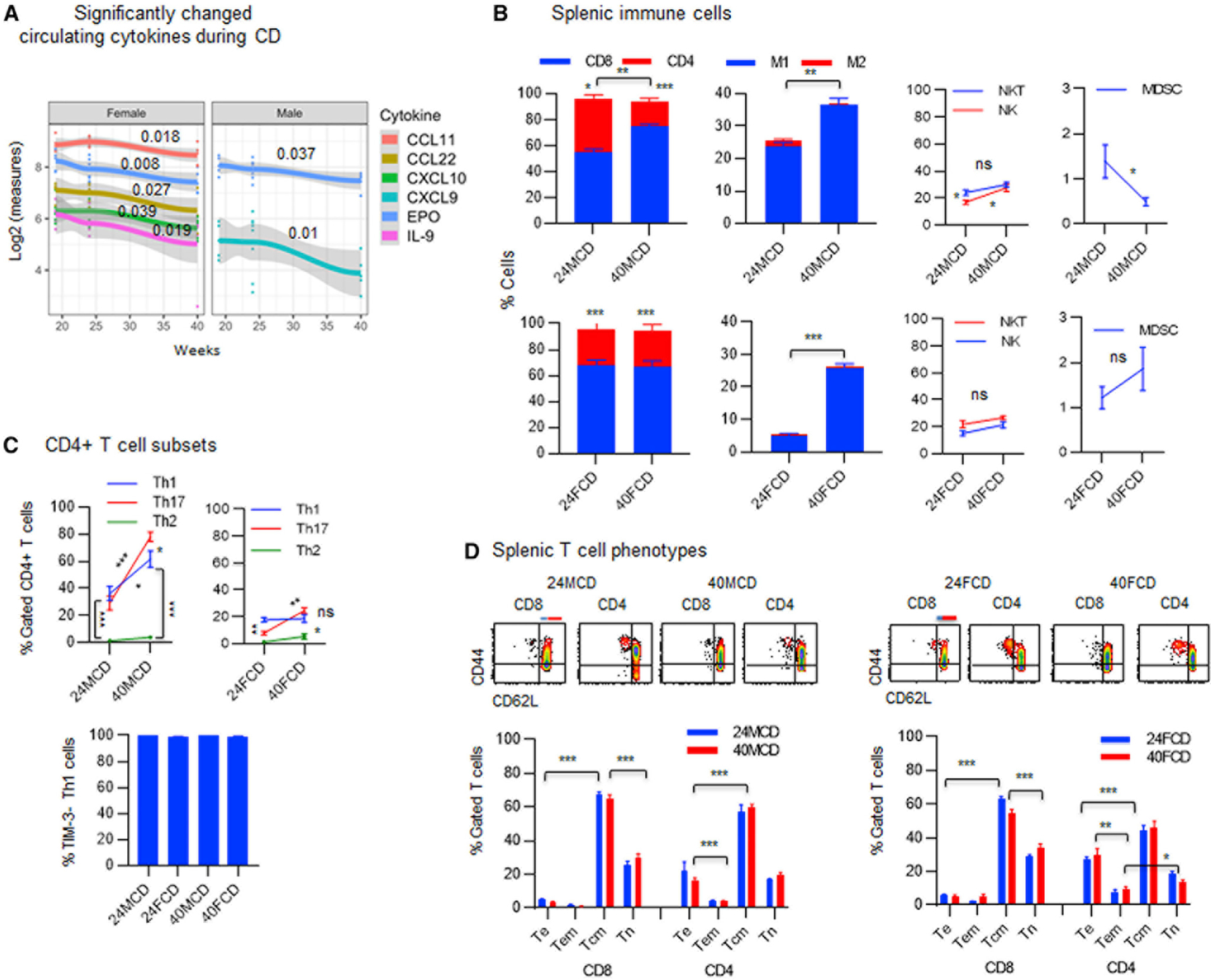
Pattern of systemic inflammation in DIAMOND mice as they age on a regular chow diet (A) Sera were collected from DIAMOND mice at weeks 19, 24, or 40 of being on a CD or WD three to five mice/group) and analyzed for 44 cytokines and chemokines in association with time using multivariate linear regression. The overall time trend, summarized across subjects, is presented for all cytokines and chemokines, which have a significant time trend during a CD. Data represent five to nine mice/group. (B) FVS^−^ viable splenocytes were gated for CD3^+^ T cells to show the proportion of CD8^+^ T cells and CD4^+^ T cells in males or females (three to five mice/group) being on a CD for 24 weeks (24MCD or 24FCD) and 40 weeks (40MCD or 40FCD) and were analyzed for M1 (F4/80^+^CD68^+^CD206^−^) and M2 macrophages (F4/80^+^CD68^+/−^CD206^+^), NK cells (CD3^−^CD4^−^CD8^−^CD49b^+^), NKT cells CD3^+^CD4^−^CD8^−^CD49b^+^), and CD11b^+^Gr1^+^ MDSCs. (C) FVS^−^ viable splenocytes gated for CD3^+^CD4^+^ T cells were analyzed for the percentage of Th1 (CXCR3^+^CCR5^−^), Th2 (CCR8^+^IL-3Ra^−^), or Th17 (CCR6^+^CD161^−^) cells: percentage of Th1 cells that do not express TIM-3 was determined. (D) FVS^−^ viable splenic CD8^+^ or CD4^+^ T cells were analyzed for percentage of T cell phenotypes, including Te (CD44^+^CD62L^−^), Tem (blue line, CD44^+^CD62L^low^), Tcm (red line, CD44^+^CD62L^high^), and Tn (CD44^−^CD62L^+^) cells. Error bars are SEM.

Animals were studied during aging on a CD in order to determine whether a higher incidence of NAFLD progression to HCC in male DIAMOND mice was because of the gender-associated inflammation or host-protective immune surveillance, regardless of a WD. Among 44 inflammatory cytokines and chemokines in the serum, females showed significantly decreasing trends for four pro-inflammatory cytokines and chemokines (CCL11, CCL22, CXCL10, and interleukin-9 [IL-9]) while males showed a significantly decreasing trend only for the pro-inflammatory CXCL9 (Figure 2A). A decreasing trend for the anti-inflammatory erythropoietin (EPO) was detected in males and females (Figure 2A). Analysis of the splenic immune cells showed that aging was associated with a significantly increasing trend (**p = 0.001) toward the CD8^+^ > CD4^+^ pattern in males (***p < 0.0001), while females sustained the CD8^+^ > CD4^+^ pattern (***p < 0.0001; Figure 2B). A significantly increased M1 > M2 pattern was evident in males and females (Figure 2B; **p = 0.001; ***p < 0.0001). Males showed shifts from the NKT > NK and Th1 = Th17 > Th2 patterns to NKT = NK and an inflammatory Th17 > Th1 > Th2 patterns because of significantly increasing Th17 (***p < 0.0001) and Th1 (*p = 0.01), while females sustained the NKT = NK pattern and switched from the Th1 > Th17 > Th2 to Th1 = Th17 > Th2 pattern as they aged on CD (Figure 2C). All Th1 cells were negative for the inhibitory receptor TIM-3 (Figure 2C). A predominant Tcm > Te pattern was sustained for the splenic CD8^+^ or CD4^+^ T cells in males and females during aging (Figure 2D; ***p < 0.0001).

### Male and female mice exhibit distinct hepatic inflammatory patterns, with males dominating the inflammatory Th17 > Th1 > Th2, NKT > NK pattern and females modulating the equilibrium Th1 = Th17 = Th2, NKT = NK pattern as they age on a CD

**Figure 3.**
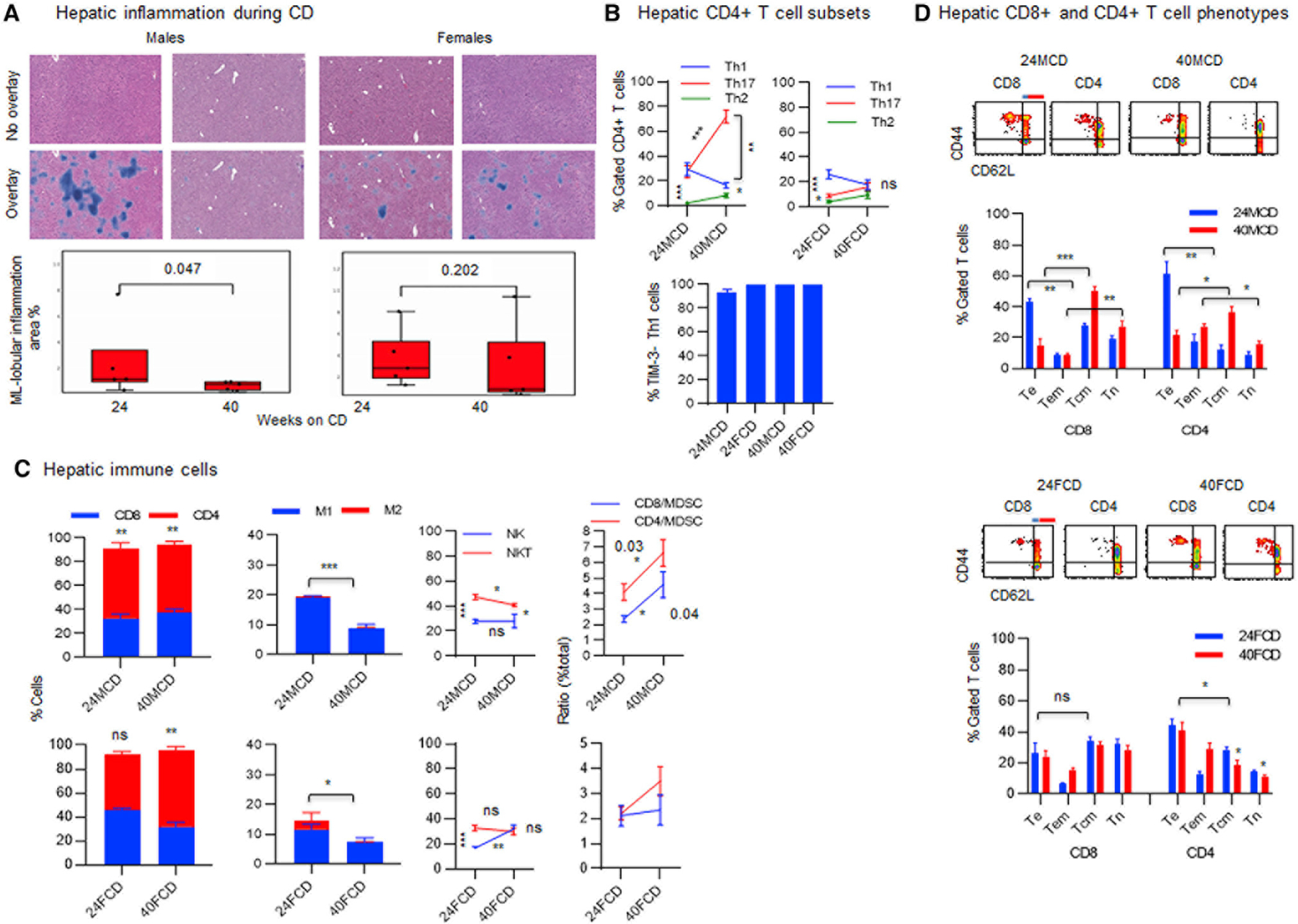
Females tend to modulate the hepatic inflammation better than males as they age on a regular chow diet (A) Female or male DIAMOND mice received a CD for 24 and 40 weeks, starting at 10 weeks of age (three to five mice/group). Livers were collected after 24 and 40 weeks of being on a CD and subjected to an H&E staining followed by enumeration of inflammatory cells using a machine learning (ML) model trained to detect and quantitate lobular inflammation on whole-slide histology images. ML lobular inflammation is shown as blue color. (B) FVS^−^ viable cells gated for CD3^+^CD4^+^ T cells were analyzed for the percentage of Th1 (CXCR3^+^CCR5^−^), Th2 (CCR8^+^IL-3Ra^−^), or Th17 (CCR6^+^CD161^−^) cells: percentage of Th1 cells that do not express TIM-3 was determined. (C) FVS^−^ viable cells were gated for CD3^+^ T cells to show the proportion of CD8^+^ T cells and CD4^+^ T cells in males or females being on a CD for 24 weeks (24MCD or 24FCD) and 40 weeks (40MCD or 40FCD), were analyzed for M1 (F4/80^+^CD68^+^CD206^−^) and M2 macrophages (F4/80^+^CD68^+/−^CD206^+^), and were analyzed for NK cells (CD3^−^CD4^−^CD8^−^CD49b^+^) and NKT cells (CD3^+^CD4^−^CD8^−^CD49b^+^). Percent total cells were analyzed for the ratio of CD8^+^ T cells to MDSC (CD8/MDSC) or CD4^+^ T cells to MDSC (CD4/MDSC). (D) FVS^−^ viable splenic CD8^+^ or CD4^+^ T cells were analyzed for percentage of T cell phenotypes, including Te (CD44^+^CD62L^−^), Tem (blue line, CD44^+^CD62L^low^), Tcm (red line, CD44^+^CD62L^high^), and Tn (CD44^−^CD62L^+^) cells. Error bars are SEM. *p ≤ 0.01, **p ≤ 0.001, and ***p ≤ 0.0001.

As animals aged on a CD, infiltrating immune cells or lobular inflammation was significantly decreased in males only (Figure 3A; p = 0.047). Analysis of the hepatic inflammatory cells showed a shift from the Th17 = Th1 > Th2 pattern by significantly increasing Th17 cells (***p = 0.0003) toward an inflammatory Th17 > Th1 > Th2 pattern in males (Figure 3B; **p = 0.001). On the other hand, females showed a shift from the Th1 > Th17 > Th2 pattern toward an equilibrium Th1 = Th17 = Th2 pattern as they aged on a CD (Figure 3B). All Th1 cells were negative for the inhibitory receptor TIM-3 (Figure 3B). During aging, the hepatic M1 > M2 pattern significantly decreased in males and females (Figure 3C; ***p < 0.0001 and *p = 0.03). Analysis of the immune cells that are associated with the hepatic immune surveillance showed a sustained CD4^+^ > CD8^+^ pattern in males (**p = 0.002), while females showed a shift from an equilibrium CD4^+^ = CD8^+^ pattern (p = not significant [ns]) toward predominant CD4^+^ > CD8^+^ pattern (**p = 0.003) during aging (Figure 3C). The M1 > M2 pattern showed a significantly decreasing trends in males and females (Figure 3C). Also, males retained NKT > NK pattern while females shifted from the NKT > NK pattern to an equilibrium NKT = NK pattern (Figure 3C). Because of predominant hepatic CD4^+^ T cells, the suppressive ratio of T cells and myeloid-derived suppressor cells (MDSCs) (smaller than 1) was analyzed on percent total cells. The ratio of T cells to MDSCs remained greater than one in males and females (Figure 3C). We have reported that the T cell and MDSC ratio of smaller than one is suppressive (Morales et al., 2010). While males showed shifts from predominant CD8^+^ and CD4^+^ Te > Tcm patterns (Figure 3D; **p = 0.003 and **p = 0.001) toward predominant CD8^+^ and CD4^+^ Tcm > Te patterns (Figure 3D; ***p = 0.0002 and *p = 0.01), females sustained an equilibrium CD8^+^ Te = Tcm and a predominant CD4^+^ Te > Tcm (*p = 0.01) pattern during aging (Figure 3D).

### Female and male mice display acute and chronic patterns with distinct systemic inflammatory cytokines during tumor progression on a WD

In order to determine the trend of systemic inflammation during WD, 44 cytokines and chemokines were analyzed in the sera of animals over time. By 40 weeks of being on a WD, four inflammatory cytokines and chemokines (CXCL9, IL-2, LIF, and TIMP1) showed significant changes, with females exhibiting an acute pattern of response during WD by significantly raising at week 19 and subsequent drop compared with those during CD (Figure S3A, upper panels). In contrast, these cytokines and chemokines showed chronic patterns in males by continuously increasing or persisting during WD (Figure S3A, lower panels). By 48 weeks of being on WD, distinct patterns of inflammatory cytokines and chemokines were detected in males and females (Figure S3B). Specifically in females, seven cytokines and chemokines significantly increased (granulocyte-macrophage colony-stimulating factor [GM-CSF], p = 0.001; IL-13, p = 0.04; KC/CXCL1, p = 0.04; LIX/CXCL5, p = 0.004; MIP-1α/CCL3, p = 0.004; MIP-1β/CCL4, p = 0.0004; TIMP-1, p = 0.04) and four cytokines and chemokines significantly decreased (CCL22, p = 0.0004; EPO, p < 0.0001; IL-11, p = 0.04; MIP-3β/CCL19, p = 0.03) during HCC. In males, discordantly three cytokines and chemokines increased (IL-12 p40, p = 0.002; LIX/CXCL5, p = 0.003; MIP-1β/CCL4, p = 0.02) and four cytokines and chemokines decreased (IL-20, p = 0.02; M-CSF, p = 0.04; MIP-3β/CCL19, p = 0.008; EPO, p = 0.004) during HCC (Figure S3B). Among 44 inflammatory cytokines and chemokines, MCP-1/CCL2 was found to have a prognostic value in females, but not in males, by a significant increase during the progression of HCC (Figure S3C; p = 0.05) and a significant decrease during the rescue from HCC (Figure S3B; p = 0.05). Similarly, analysis of 65 cytokines and chemokines in the sera of patients during the progression of NASH versus healthy controls showed significantly decreased CXCL1 (p = 0.05) and CXCL12 (p = 0.03) as well as significantly increased CCL7 (p = 0.03), CXCL10 (p = 0.001), IL-1Rα (p = 0.001), IL-33 (p = 0.02), IL-16 (p = 0.02), and IL-28α (p = 0.02) only in women during NASH (Figure S4). Men with NASH did not show significant changes in inflammatory cytokines and chemokines compared with healthy controls (Figure S4).

### The predominant hepatic CD8^+^ > CD4^+^, NKT > NK, M1 > M2 pattern is associated with tumor progression, whereas an equilibrium CD8^+^ = CD4^+^, NKT = NK, M1 = M2 pattern is associated with distinct tumor inhibition in a gender-specific manner

**Figure 4.**
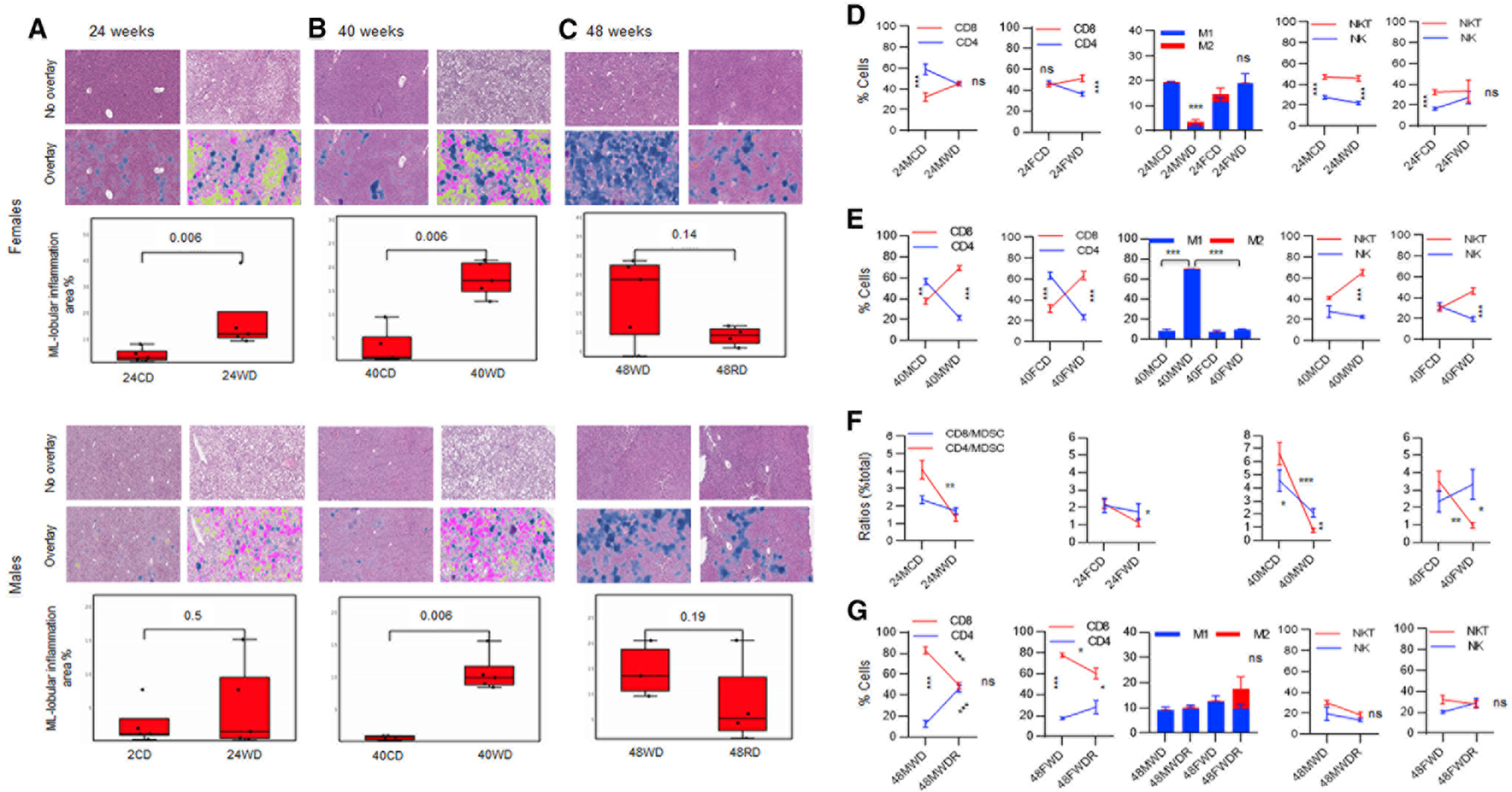
The hepatic immune responses during tumor progression or a rescue from HCC (A) Female or male DIAMOND mice received a CD or WD for 24 (24FCD, 24FWD, 24MCD, and 24MWD) and 40 weeks, starting at 10 weeks of age (three to five mice/group). A group of female or male mice set aside to receive a WD for 48 weeks (48FWD or 48MWD) or being on a WD for 36 weeks and receiving a diet reversal to a CD for an additional 12 weeks through week 48 (48MWDR and 48FWDR) is shown. (A–C) Livers were collected after 24, 40, and 48 weeks of being on diets and subjected to an H&E staining followed by enumeration of inflammatory cells using a ML model trained to detect and quantitate lobular inflammation on whole-slide histology images. ML lobular inflammation is shown as blue color. Macrosteatosis and microsteatosis are shown as pink and yellow colors, respectively. (D and E) FVS^−^ viable hepatic cells gated for CD3^+^ T cells and analyzed for the percentage of CD8^+^ and CD4^+^ T cells, M1/2 cells, and NKT/NK cells in females or males on a CD or a WD for 24 weeks (24MCD, 24FCD, 24MWD, and 24FWD) or 40 weeks (40MCD, 40FCD, 40MWD, and 40FWD). (F) Percent total cells were analyzed for the ratio of CD8^+^ T cells to MDSC (CD8/MDSC) or CD4^+^ T cells to MDSC (CD4/MDSC). (G) The hepatic T cells, M1/M2 macrophages, and NKT/NK cells were analyzed in males and females after 48 weeks of being on WD (48MWD and 48FWD) as well as those on a diet reversal (48MWDR and 48FWDR). Error bars are SEM. *p % 0.01, **p % 0.001, and ***p % 0.0001.

A comparative analysis of lobular inflammation during WD versus CD showed earlier infiltrating immune cells in the liver of females compared with those in males at week 24 (Figure 4A; p = 0.006). Males showed a late lobular infiltration at week 40 during WD (Figure 4B; p = 0.006), when females sustained lobular infiltration through weeks 24 and 40 (Figure 4B, p = 0.006). Reversal of WD to CD resulted in insignificant decline in hepatic immune cells in males or females (Figure 4C). In order to determine the cellular component of lobular inflammation, hepatic immune cells were analyzed during WD compared with those during CD. An earlier lobular inflammation in females during WD contained different immunological patterns compared with males, with females showing a shift from an equilibrium CD8^+^ = CD4^+^ pattern to a predominant CD8^+^ > CD4^+^ pattern inversely correlated with their patterns in males (Figure 4D; ***p = 0.003). Also, females sustained M1 > M2 pattern and switched from NKT > NK to NKT = NK pattern while males significantly decreased M1 > M2 pattern and sustained NKT > NK pattern (Figure 4D). Males and females showed similar patterns of these immune cells in the spleen (Figure S5A).

After 40 weeks of being on a WD when HCC was first detected, a shift from predominant hepatic CD4^+^ > CD8^+^ pattern to a predominant CD8^+^ > CD4^+^ pattern was evident in males and females (Figure 4E; **p = 0.002; ***p < 0.0001–p = 0.0003). The hepatic M1 > M2 pattern was significantly increased in males during WD compared with that during CD or compared with females showing no changes in the M1 > M2 pattern (Figure 4E). The equilibrium NKT = NK pattern shifted to a predominant NKT > NK pattern in the liver of both males and females (Figure 4E). Again, splenic patterns of these immune cells were comparable in males and females (Figure S5B; *p = 0.01; ***p = 0.0003). Being on a WD for 24 weeks significantly reduced the ratio of the hepatic CD4^+^ T cells to MDSCs in males and females without reaching below a 1:1 ratio to be suppressed (Figure 4F, upper panels). The ratio of the splenic CD4^+^ T cells to MDSCs did not change, with females showing a significantly decreased ratio of CD8^+^ T cells to MDSC, but the ratio remained greater than 1:1 (Figure S5C, upper panels). However, during tumor development at 40 weeks of being on a WD, the ratio of the hepatic CD4^+^ and CD8^+^ T cells to MDSC significantly decreased in males, while females only decreased the CD4^+^ T cells/MDSC ratio, with the CD4^+^ T cells/MDSC ratio lowering below a 1:1 ratio in males and females (Figure 4F, lower panels). The splenic T cells remained higher than MDSC at this time (Figure S5C, lower panels).

Reversal of WD to CD resulted in a shift from predominant CD8^+^ > CD4^+^ pattern to an equilibrium CD8^+^ = CD4^+^ pattern in male and sustained predominant CD8^+^ > CD4^+^ pattern in females, though both males and females showed significantly decreasing trends for CD8^+^ T cells (Figure 4G; ***p < 0.0001 and *p = 0.01). While males sustained a predominant M1 > M2 pattern during diet reversal, females switched from a predominant M1 > M2 to an equilibrium M1 = M2 pattern because of a significant increase in M2 cells (***p < 0.0001; Figure 4G). An equilibrium pattern of the hepatic NKT = NK cells was evident in males and females (Figure 4G). In the spleen, the CD8^+^ > CD4^+^ pattern remained predominant in males (***p < 0.0001), while females shifted to an equilibrium CD8^+^ = CD4^+^ pattern (Figure S5D). No changes were detected in the splenic M1 > M2 or NKT = NK patterns during diet reversal (Figure S5D).

### The hepatic CD8^+^ and CD4^+^ Te > Tcm patterns become predominant during a WD, as well as during the progression or inhibition of HCC

**Figure 5.**
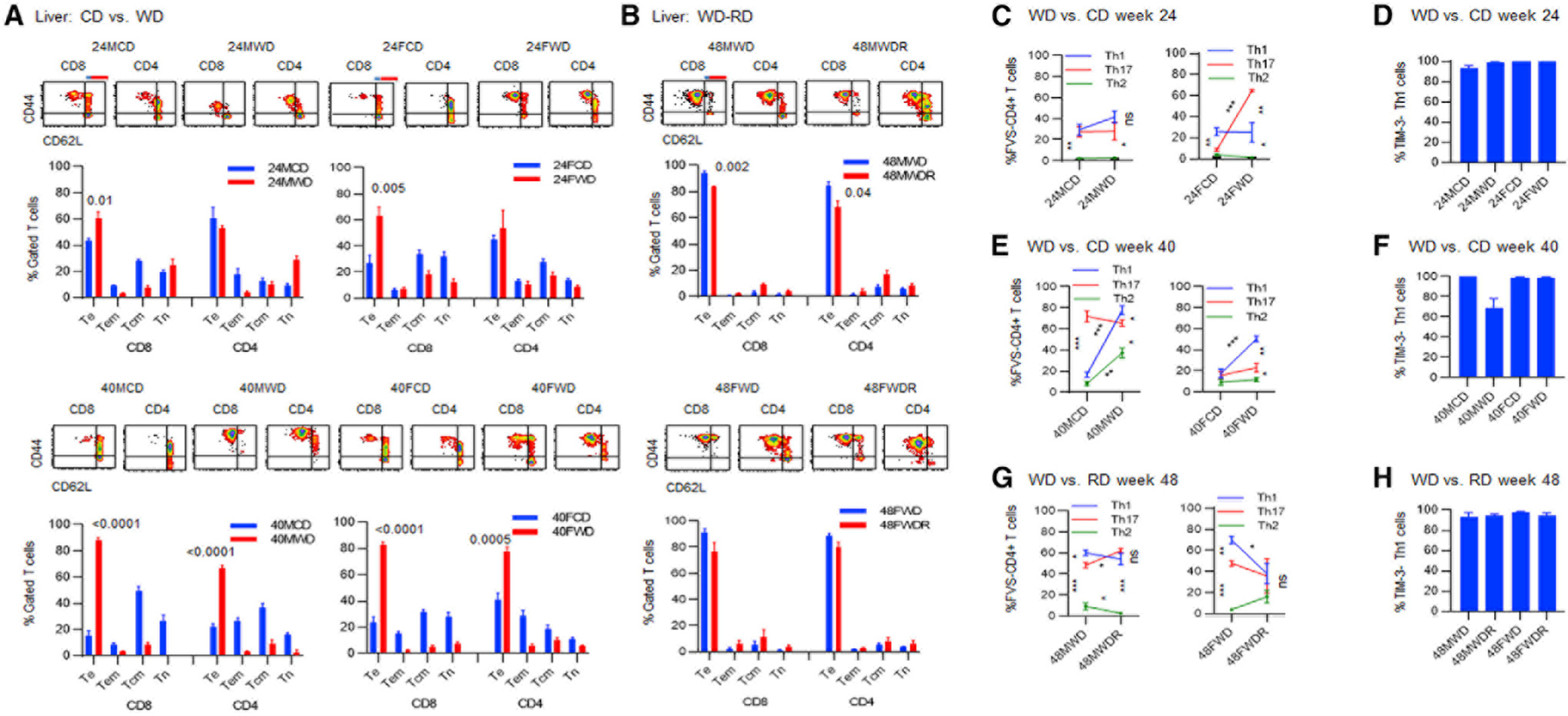
Pattern of the hepatic CD8^+^ and CD4^+^ phenotypes and Th1, Th2, and Th17 cells during the progression of or a rescue from HCC Female or male DIAMOND mice were fed a CD or a WD for 24 weeks (24FCD, 24FWD, 24MCD, and 24MWD) and 40 weeks, starting at 10 weeks of age. A group of female or male mice set aside to receive a WD for 48 weeks (48FWD or 48MWD) or put back on a CD at 36 weeks of being on a WD and continued on a CD for an additional 12 weeks through week 48 (48MWDR and 48FWDR) is shown. (A and B) FVS^−^ viable hepatic CD8^+^ or CD4^+^ T cells were analyzed for percentage of Te (CD44^+^CD62L^−^), Tem (blue line, CD44^+^CD62L^low^), Tcm (red line, CD44^+^CD62L^high^), and Tn (CD44^—^CD62L^+^) cells. (C) FVS^−^ viable hepatic cells gated for CD4^+^ T cells were analyzed for the percentage of Th1 (CD3^+^CD4^+^CXCR3^+^CCR5^−^), Th2 (CCR8^+^IL-3Ra^−^), or Th17 (CD3^+^CD4^+^CCR6^+^CD161^−^) cells after 24 weeks of being on a CD or a WD. (D) The hepatic Th1 cells were analyzed for the expression of TIM-3. (E) FVS^−^ viable hepatic cells gated for CD4^+^ T cells were analyzed for the percentage of Th1 or Th17 cells after 40 weeks of being on a CD or a WD. (F) The hepatic Th1 cells were analyzed for the expression of TIM-3. (G) FVS^−^ viable hepatic cells gated for CD4^+^ T cells were analyzed for the percentage of Th1, Th2, or Th17 cells after 48 weeks of being on a WD or during an RD. (H) The hepatic Th1 cells were analyzed for the expression of TIM-3, after 48 weeks of being on a WD or during an RD. Data represent three to five mice/group. Error bars are SEM. *p % 0.01, **p % 0.001, and ***p % 0.0001.

Analysis of the hepatic T cell phenotypes showed predominant CD8^+^ and CD4^+^ Te > Tcm patterns in males and females after 24 weeks of being on a WD (Figure 5A). During this period, the splenic CD8^+^ and CD4^+^ T cells in males showed a shift from predominant Tcm > Te pattern toward predominant Tn > Te pattern whereas those in females sustained a predominant Tcm > Te pattern (Figure S6A). After 40 weeks of being on a WD, predominant hepatic CD8^+^ and CD4^+^ Te > Tcm patterns sustained in the liver (Figure 5A, lower panels) and in the spleen (Figure S6A, lower panels). During a diet reversal and rescue from HCC, the hepatic CD8^+^ and CD4^+^ Te cells declined in males, but the CD8^+^ and CD4^+^ Te > Tcm patterns sustained in males and females (Figure 5B). Similar trends were detected in the splenic T cell phenotypes with only males showing significantly reduced CD4^+^ Te phenotype during a diet reversal (Figure S6B; ***p < 0.0001).

### The predominant Th1 inflammatory pattern, Th1 > Th17 > Th2, is associated with tumor progression, whereas semi-equilibrium Th1 = Th17 > Th2 and equilibrium Th1 = Th17 = Th2 patterns are, respectively, associated with a partial and a complete rescue from HCC

In order to determine whether a better protection of females from HCC was associated with the modulation of inflammatory Th cell patterns, the hepatic and splenic Th cells were analyzed. Within 24 weeks of being on a WD, males showed no changes in the hepatic Th1 = Th17 > Th2 pattern while females significantly increased Th17 cells (Figure 5C; ***p < 0.0001), shifting from a predominant Th1 pattern, Th1 > Th17 = Th2, to a predominant Th17 pattern, Th17 > Th1 > Th2 pattern (Figure 5C; **p = 0.003). No changes were detected in the level of the hepatic’TIM-3^−^ Th1 cells (Figure 5D). During this time, the splenic Th1 = Th17 > Th2 pattern was detected in males and females, with only females showing significantly increasing trends for Th1 and Th17 (Figure S7A; ***p < 0.0001 and **p = 0.001). No changes were detected in the level of the splenic TIM-3^−^ Th1 cells (Figure S7B). At 40 weeks of being on a WD and during tumor development, both males and females significantly increased the hepatic Th1 cells (Figure 5E; ***p < 0.0001), exhibiting a predominant Th1 inflammatory pattern, Th1 > Th17 > Th2. During this period, the splenic Th1 = Th17 > Th2 pattern was detected in males and females (Figure S7C). Partial rescue of males from HCC following a diet reversal was associated with significantly increasing the hepatic Th17 cells (Figure 5G; *p = 0.01) and shifting from a predominant Th1 pattern (Th1 > Th17 > Th2) toward an inflammatory Th17 by establishing the Th1 = Th17 > Th2 pattern (Figure 5G; *p = 0.01) while sustaining a Th1 inflammatory pattern, Th1 > Th17 > Th2, in the spleen (Figure S7E; **p = 0.003). Complete protection of females following a diet reversal was associated with the establishment of an equilibrium Th1 = Th17 = Th2 pattern in the liver (Figure 5G; *p = 0.01) and in the spleen (Figure S7E).

## DISCUSSION

Men show a higher incidence with severe manifestations of NAFLD and NASH and HCC than women (Lonardo et al., 2019; Natarajan et al., 2020). This disparity is thought to be due to the ability of women to resolve inflammation and inhibit chronic inflammation better than men (Rathod et al., 2017). In fact, women produce higher levels of specialized pro-resolving mediators (SPMs), such as lipoxins, protectins, resolvins, and maresins (Rathod et al., 2017). Paradoxically, women are four times more likely to develop autoimmune hepatitis than men (Manns et al., 2010). Therefore, these disparities cannot be fully explained in the context of inflammation without understanding of the adaptive immune response in the liver. Genetically, the X chromosome is suggested to play key role in the immune response since many proteins that are involved in immune responses are encoded on the X chromosome (Libert et al., 2010). For instance, Toll-like receptors (TLRs), CD40 ligand, and the main proteins associated with nuclear factor kB (NF-kB) signaling pathway are linked to the X chromosome (Spolarics, 2007). Although one of the two X chromosomes in females is randomly inactivated by methylation, about 15% of X-linked genes escape methylation, resulting in increased proteins linked to the X chromosome in women compared with men (Carrel and Willard, 2005; Chitnis et al., 2000). In other words, females are composed of a mosaic of cells from paternal and maternal X chromosomes, providing them with greater diversity of immune responses (Spolarics, 2007; Spolarics et al., 2017) and enabling them to show lower levels of some inflammatory cytokines and better immune responses compared with men (Conti and Younes, 2020).

To decipher how tumor progression or inhibition occurs in a gender-specific manner during inflammatory NAFLD, we took a perturbation approach (Paus, 2005) and discovered distinct immunological patterns during the disease progression or inhibition. This pattern discovery approach has recently been introduced for the understanding of tumor dormancy and relapse (Manjili and Khazaie, 2022). Here, we demonstrated that, similar to humans, female DIAMOND mice had a lower incidence of NAFLD-associated HCC. A lower susceptibility of female mice to NAFLD was associated with distinct anatomical deposition of fat predominantly in the abdominal region compared with males showing fat deposition mainly in the liver. Also, female mice responded to a corrective diet better than males by a complete rescue from steatohepatitis and HCC.

Longitudinal studies during aging on a CD revealed gender predisposition of males to NAFLD-associated HCC. In fact, females were able to modulate aging-associated several inflammatory cytokines compared with males. In the liver, females tend to modulate the hepatic inflammation by establishing the Th1 = Th17 = Th2 and NKT = NK patterns while males tend to mount the inflammatory Th17 > Th1 > Th2 and NKT > NK patterns during aging on a CD. In addition, females tend to sustain predominant hepatic CD4^+^ and CD8^+^ Te phenotypes, while males tend to dominate CD4^+^ and CD8^+^ Tcm phenotypes during aging (Table 1). A stronger hepatic immune system by sustaining Te phenotypes while maintaining an equilibrium Th1 = Th17 = Th2, NKT = NK pattern in the liver of females could explain their lower susceptibility to NAFLD and HCC as well as their higher susceptibility to autoimmune liver disease compared with males. In response to a WD, females mounted an early and acute pattern of the inflammatory cytokines CXCL9, LIF, IL-2, and TIMP-1, whereas males mounted a delayed and chronic pattern of these cytokines. An acute versus a chronic pattern of inflammation is important because of short-term anti-fibrotic and long-term pro-fibrotic effects of CXCL9 (Berres et al., 2015; Tacke et al., 2011; Wasmuth et al., 2009) and TIMP-1 (Thiele et al., 2017; Wang et al., 2011). Although IL-2 supports the adaptive immune response, a chronically increased IL-2 could induce T cell apoptosis.

During the progression of NAFLD and HCC, females elevated systemic inflammatory responses by increasing and sustaining the splenic Th1 and Th17 cells while males showed declined splenic Th1 and Th17 cells, though an equilibrium splenic Th1 = Th17 pattern was sustained in male and female mice. Also, females elevated seven inflammatory cytokines (GM-CSF, IL-13, CXCL1, CXCL5, CCL3, CCL4, and TIMP-1), while males increased only three inflammatory cytokines IL-12, CXCL5, and CCL4. Similar trends were detected in patients with NASH, with women, but not men, elevating six inflammatory cytokines IL-1Ra, CXCL10, IL-16, CCL7, IL-33, and IL-28α. These data suggest that circulating inflammatory cytokines might have a prognostic value in females, but not in males, during the progression of NAFLD and NASH.

Tumor progression in both males and females was associated with the hepatic inflammatory patterns CD8^+^ >CD4^+^, Th1 > Th17 > Th2, NKT > NK, and M1 > M2 (Table 2). A complete and a partial rescue from HCC were associated with a complete and a partial resolution of the hepatic inflammatory patterns in females and males, respectively. Following a diet correction, females established an equilibrium Th1 = Th17 = Th2, NKT = NK, M1 = M2 pattern in the liver as well as an equilibrium NKT = NK and a semi-equilibrium Th1 = Th17 > Th2 pattern in the spleen, while males established a semi-equilibrium Th1 = Th17 > Th2 and an equilibrium NKT = NK pattern but sustained an inflammatory M1 > M2 pattern in the liver (Table 2). Also, females sustained an anti-tumor CD8^+^ > CD4^+^ pattern while males shifted to a CD8^+^ = CD4^+^ pattern in the liver (Table 2). We did not include T regulatory cells (Treg cells) in the pattern recognition of Th cells because changes in the hepatic Treg cells were negligible. Furthermore, contradictory findings have been reported on Treg cells detected in the visceral fat, blood, or liver samples of patients with NASH as well as in animal models (Van Herck et al., 2019; Ma et al., 2018).

These data suggest that predominant Th1, but not Th17, inflammatory pattern promotes HCC. Our findings are consistent with other reports showing a higher frequency of interferon γ (IFN-γ)-producing Th1 cells during the progression of NAFLD to NASH (Rau et al., 2016). IFN-γ has also been reported to be involved in the initiation stage of diethylnitrosamine-induced HCC due to its inflammatory function (Matsuda et al., 2005) and during NAFLD (Alisi et al., 2017; Luo et al., 2017). Also, CD4^+^ Th1 cells are reported to be involved in obesity-related insulin resistance (Cho et al., 2014). On the other hand, anti-tumor function of Th17 has been supported in a systemic review of literature on cancer, showing that Th17 cells are associated with improved prognosis while IL-17 is associated with poor prognosis (Punt et al., 2015). In fact, IL-17-producing Th17 cells are reported to exacerbate tumorigenesis and liver damage (Chang, 2019), while IL-22-producing Th17 cells could facilitate recovery of the liver from injury (Arshad et al., 2020). In regard to macrophages, M1 macrophages, while displaying anti-tumor function, could also cause liver damage (Zimmerer et al., 2018). Very recent analysis revealed that M1 macrophages with an M1 > M2 pattern were associated with aggressive cancer biology without any survival benefit (Oshi et al., 2020). Also an M1 > M2 pattern promoted NAFLD-associated HCC (Wu et al., 2020). Our data also suggest that the hepatic CD8^+^ and CD4^+^ Te phenotypes might initially have anti-tumor function but could promote tumor growth when they remain chronically elevated throughout a WD. This possibility is supported by very recent observations in non-viral HCC such that increased hepatic CD8^+^ T cells resulted in increased incidence of liver damage, NASH, and HCC in preclinical models as well as in patients with NASH (Pfister et al., 2021). It is also reported that CD8^+^ T cells can increase glucose uptake and glycolysis after activation (Macintyre et al., 2014).

Based on our findings, we propose that identification of the immunological patterns rather than pinpointing specific immune cells could better explain the immunobiological complexity of HCC. This is consistent with emerging data demonstrating that imbalance of Th cells promotes NAFLD and chronic liver diseases (He et al., 2017; Zhang et al., 2021). A very recent report demonstrated that patterns of dendritic cells and monocyte subsets are associated with the severity and mortality of patients with liver cirrhosis (Cardoso et al., 2021). In fact, an equilibrium Th1 = Th17 = Th2, NKT = NK, M1 = M2 pattern; semi-equilibrium Th1 = Th17 > Th2, CD8^+^ = CD4^+^, NKT = NK pattern; or predominant CD8^+^ > CD4^+^, Th1 > Th17 > Th2, NKT > NK, M1 > M2 pattern could each exhibit distinctively collective functions independent from the cellular components. This is because of the counter-regulatory role of cytokines released by these cells. For instance, Th1 cytokines inhibit Th2 cells vice versa, resulting in the formation of different functional entities when these cells display in equilibrium or dominant pattern.

### Limitations of the study

We went beyond a two-dimensional cause-effect analysis of the immune response acting on tumor cells by performing a three-dimensional analysis of immune cell types, their interactions, and functional changes over time. However, identification of a collective functional attributes of immunological patterns, which could be independent from the function of each component, would require a four-dimensional analysis. This would be possible by deep sequencing at a single-cell level encompassing the hepatic immune cell types, network of their interactions, gene regulations, and functional changes over time. Nevertheless, our findings through a perturbation approach (Paus, 2005) sets the stage for generating the hypothesis for more in-depth studies, including discovery of pattern-derived functional entities using a single-cell RNA sequencing (RNA-seq) approach.

## STAR*METHODS

### RESOURCE AVAILABILITY

#### Lead contact

All information and requests should be directed to the lead contact, Masoud Manjili (masoud.manjili@vcuhealth.org)

#### Materials availability

DIAMOND mice used in this study will be provided, upon request to the lead contact, and may require fulfillment of an MTA. This study did not generate new unique reagents.

#### Data and code availability

- Microscopy data reported in this paper will be shared by the lead contact upon request
- This paper does not report original code.
- Any additional information required to reanalyze the data reported in this paper is available from the lead contact upon request

### EXPERIMENTAL MODEL AND SUBJECT DETAILS

#### Animals

Two month old DIAMOND female and male mice were put on a standard chow diet (CD; Harlan TD.7012) or high-fat, high-carbohydrate diet (Harlan TD.88137) containing 42% kcal from milk fat and 0.1% cholesterol, and drinking water containing a high fructose (23.1 g/L) and glucose (18.9 g/L) (WD) (Asgharpour et al., 2016) for 48–60 weeks. Sera were collected at 19, 24, and 40 weeks of being on CD or WD for multiplex cytokine analysis. A reverse diet group (RD) were put on CD after 36 weeks of being on WD, when animals developed progressive NASH (Asgharpour et al., 2016), and were followed by weeks 48 through 60. Animals were sacrificed at weeks 24, 40 or 48–60 of being on diet, and their livers and spleens were subjected to multiplex flow cytometry analysis of immune cells as well as IHC analysis of fatty liver and HCC. Five mice per group were used unless stated otherwise. The time points were chosen because of our previous observations on clinical manifestation of NAFLD (24 weeks), progressive NASH (36–40 weeks) and stage III HCC (48–60 weeks) (Asgharpour et al., 2016). These studies have been reviewed and approved by the Institutional Animal Care and Use Committee at Virginia Commonwealth University.

### METHOD DETAILS

#### Multiplex cytokine array

Mouse Cytokine 44-Plex Discovery Assay (Eva Technologies, Calgary, AB, Canada) was used to detect 44 cytokines in the sera. Cytokines/chemokine panel included Eotaxin, Erythropoietin, 6Ckine/CCL21, Fractalkine, G-CSF, GM-CSF, IFN-β1, IFN-γ, IL-1α, IL-1β, IL-2, IL-3, IL-4, IL-5, IL-6, IL-7, IL-9, IL-10, IL-11, IL-12 (p40), IL-12 (p70), IL-13, IL-15, IL-16, IL-17, IL-20, IP-10/CXCL10, KC/CXCL1, LIF, LIX/CXCL5, MCP-1/CCL2, MCP-5/CCL12, M-CSF, MDC/CCL22, MIG/CXCL9, MIP-1α/CCL3, MIP-1β/CCL4, MIP-2/CXCL2, MIP-3α/CCL20, MIP-3β/CCL19, RANTES/CCL5, TARC/CCL17, TIMP-1, TNFα, and VEGF. Human Cytokine/Chemokine 65-Plex Discovery Assay (Eva Technologies, Calgary, AB, Canada) was used to detect 65 cytokines in the sera of healthy male control (MC) or female control (FC) as well as males or females with NASH (MNASH and FNASH). Cytokines/chemokine panel included EGF, Eotaxin, FGF-2, Flt-3 ligand, Fractalkine, G-CSF, GM-CSF, GRO, IFN-α2, IFN-γ, IL-10, IL-12 (p40), IL-12 (p70), IL-13, IL-15, IL-17A, IL-18, IL-1 Rα, IL-1α, IL-1β, IL-2, IL-3, IL-4, IL-5, IL-6, IL-7, IL-8, IL-9, IP-10, MCP-1/CCL2, MCP-3/CCL7, MDC/CCL22, MIP-1α/CCL3, MIP-1β/CCL4, PDGF-AA, PDGF-AB/BB, RANTES/CCL5, TGF-α, TNF-α, TNF-β, VEGF, sCD40L, Eotaxin-2/CCL24, MCP-2/CCL8, BCA-1/CXCL13, MCP-4/CCL13, I-309/CCL1, IL-16, TARC/CCL17, 6CKine/CCL21, Eotaxin-3/CCL26, LIF, TPO, SCF, TSLP, IL-33, IL-20, IL-21, IL-23, TRAIL, CTACK/CCL27, SDF-1/CXCL12, ENA-78/CXCL5, MIP-1d/CCL15, IL-28A.

#### Flow cytometry and immunophenotyping

In order to perform multiparametric measurements of specific subpopulations within gated lymphoid or myeloid cells, and capture their pattern of rearrangements as gated and total frequency, flow cytometry analysis was performed. After taking small samples for formalin fixation, the whole spleen and liver collected from animals at weeks 24, 40, and 48–60 of being on a CD or a WD or a RD, were homogenized into a single cell suspension as described previously by our group (Payne et al., 2018). The Fc blocker anti-CD16/32 Ab was used for all staining panels. We used T cell phenotyping panel (CD8, CD4, CD44, CD62L), MDSC panel (CD11b, Gr1), NK/NKT cell panel (CD3, CD4, CD8, CD49b), M1/M2 panel (F4/80, CD68, CD206), Th1 panel (CD3, CD4, CXCR3, CCR5, TIM-3 or CD3, CD4, CXCR3, CCR8, CCR6, T-bet), Th17 panel (CD3, CD4, CCR6, CD161 or CD3, CD4, CXCR3, CCR8, CCR6, RoRγt), Th2 panel (CD3, CD4, CCR8, CD123 or CD3, CD4, CXCR3, CCR8, CCR6, GATA-3), and Treg panel (CD3, CD4, CXCR3, CCR8, CCR6, Foxp3). All panels were used with APC/Cy5-FVS in order to gate on FVS^—^ viable cells. All reagents were purchased from Biolegend (San Diego, CA), except for BD Horizon APC/Cy5-FVS and BUV395-CD3 (SK7) which were purchased from BD Biosciences (Franklin Lakes, NJ). We showed that CD4^+^CXCR3^+^CCR8^—^CCR6^-^ Th1 cells mainly express T-bet, but not much of other Th transcription factors (Figure S1). CCR6 is universally expressed on Th17 cells (Wang et al., 2009), which was confirmed by detecting the Th17 transcription factor RoRgt in CD4^+^CCR6^+^CCR8^−^CXCR3^-^ T cells (Figure S1). The expression of Foxp3 in the hepatic Th cells was relatively negligible during disease progression as shown by a representative data in Figure S1. All reagents were used at the manufacturer’s recommended concentration. For lymphocytes and myeloid cells, gated lymphoid and myeloid regions were analyzed, respectively. Multicolor data acquisition was performed using a LSRFortessa X-20 (BD Biosciences) and a ImageStreamX Mark II Imaging Flow Cytometer (Millipore Sigma, Billaerica, MA). Data were analyzed using FCS Express v5.0 (De Novo Software; Glendale, CA).

#### Steatosis and lobular inflammation

Formalin fixed paraffin embedded liver (FFPE) tissues were subjected to hematoxylin and eosin (H & E) stain using Tissue Tek Prisma Autostainer as previously described by our group (Idowu et al., 2012). Histology slides were scanned at 40x magnification. A previously developed machine learning (ML) algorithm (PathAI research platform; Boston, MA) quantitated key NASH features on the whole slide histology images. The ML algorithm was trained to detect steatosis, microvesicular steatosis, lobular inflammation, and hepatocellular ballooning using >100,000 expert pathologist annotations. ML-based, image level measures were obtained by computing the proportion of tissue area corresponding to each detected histologic feature.

### QUANTIFICATION AND STATISTICAL ANALYSIS

Changes in circulating cytokines were analyzed longitudinally and between groups at each time point. The ‘‘out of (upper/lower) range’’ values were replaced by the maximum/minimum measures, respectively, and other OOR values were set as missing values. The cytokine measures were log2-transformed. For the time course analysis (Figures 3A and S3B), linear regression was used to model the change in the cytokine measure over time separately for male and female subgroups. For the significant cytokine changes due to diet analysis (Figure S3A), linear regression modeling the effect of cytokine, diet, time, and their interactions were used. In the case of missing data, we use complete case analysis to exclude that time point. All analysis was performed in R version 3.6.0. p-values were corrected for multiple testing using Benjamini-Hochberg method. We also used Wilcoxon rank sum two-sided tests to compare between the various cytokine groups as desired, varying with gender and diet. Due to the small sample sizes, exact tests were used. Log-rank (Mantel-Cox) test was performed to determine progression-free survivals for two groups. For cellular analyses, statistical comparisons between groups were made using two-tailed Student’s t-test per the specific hypothesis. A *p* value % 0.05 was considered statistically significant.

## Supplementary Material

1

## Figures and Tables

**Table:** KEY RESOURCE TABLE

REAGENT or RESOURCE	SOURCE	IDENTIFIER
Antibodies		

Purified anti-mouse CD16/32	Biolegend	Cat# 101302 PRID: AB_312801
Brilliant Violet 711 anti-mouse CD8a	Biolegend	Cat# 100759 PRID: AB_2563510
APC anti-mouse CD8a	Biolegend	Cat# 100712 PRID: AB_312751
FITC anti-mouse CD4	Biolegend	Cat# 100405 PRID: AB_312690
Brilliant Violet 421 anti-mouse/human CD44	Biolegend	Cat# 103040 PRID: AB_2616903
APC anti-mouse CD62L	Biolegend	Cat# 104411 PRID: AB_313098
FITC anti-mouse/human CD11b	Biolegend	Cat# 101205 PRID: AB_312788
APC anti-mouse/human CD11b	Biolegend	Cat# 101212 PRID: AB_312795
PE anti-mouse Ly-6G/Ly-6C (Gr-1)	Biolegend	Cat# 108407 PRID: AB_313372
BUV395 hamster anti-mouse CD3e	BD Biosciences	Cat# 563565 Clone 145–2C11
PE anti-mouse CD49b	Biolegend	Cat# 103506 PRID: AB_313029
APC anti-mouse F4/80	Biolegend	PRID: AB_893493
Brilliant Violet 421 anti-mouse CD206 (MMR)	Biolegend	Cat# 141717 PRID: AB_2562232
FITC anti-mouse CD206 (MMR)	Biolegend	Cat# 141704 PRID: AB_10901166
FITC anti-mouse CD68	Biolegend	Cat# 137006 PRID: AB_10578412
PE anti-mouse CD68	Biolegend	Cat# 137014 PRID: AB_10612937
APC anti-mouse CD195 (CCR5)	Biolegend	Cat# 107011 PRID: AB_2074528
Brilliant Violet 421 anti-mouse CD366 (Tim-3)	Biolegend	Cat# 119723 PRID: AB_2616908
Brilliant Violet 421 anti-mouse CD198 (CCR8)	Biolegend	Cat# 150305 PRID: AB_2616650
PE anti-T-bet	Biolegend	Cat# 644809 PRID: AB_2028583
APC anti-mouse CD196 (CCR6)	Biolegend	Cat# 129813 PRID: AB_1877148
PE anti-mouse CD196 (CCR6)	Biolegend	Cat# 129804 PRID: AB_1279137
PE anti-mouse CD183 (CXCR3)	Biolegend	Cat# 126506 PRID: AB_1027650
Brilliant Violet 650 anti-mouse CD183 (CXCR3)	Biolegend	Cat# 126531 PRID: AB_2563160
APC anti-mouse NK-1.1	Biolegend	Cat# 108709 PRID: AB_313396
PE mouse anti-mouse RORyt	BD Biosciences	Cat# 562607 Clone Q31–378
PE anti-GATA3	Biolegend	Cat# 653803 PRID: AB_2562722
PE anti-mouse FOXP3	Biolegend	Cat# 126403 PRID: AB_1089118
Fixable Viability Stain 780	BD Biosciences	Cat# 565388 RRID:AB_2869673
PE anti-mouse CD365 (Tim-1)	Biolegend	Cat# 119506 PRID: AB_2232887
CD123 Monoclonal Antibody APC	Invitrogen	Cat# 17–1231-82 PRID: AB_891361
Mouse CXCL16 Antibody	R&D Systems	Cat# AF503
Brilliant Violet 421 anti-mouse CD186 (CXCR6)	Biolegend	Cat# 151109 PRID: AB_2616760
Brilliant Violet 421 anti-mouse CD80	Biolegend	Cat# 104725 PRID: AB_10900989
BV786 rat anti-mouse CD45	BD Biosciences	Cat# 564225 Clone 30-F11

Critical commercial assays		

Mouse Cytokine 44-Plex Discovery Assay	Eva Technologies	N/A
Human Cytokine/Chemokine 65-Plex Discovery Assay	Eva Technologies	N/A
Transcription Factor Buffer Set	BD Biosciences	Cat# 562574; RRID:AB_2869424
Deposited data		
Raw and analyzed data	This paper	CELL-REPORTS-D-21–04608R2

Experimental models: Organisms/strains		

DIAMOND mice	Dr. Arun Sanyal; Asgharpour et al., 2016	N/A

Software and algorithms		

FCS Express v5.0	De Novo Software	https://denovosoftware.com/
Machine Learning (ML) algorithm	PathAI research platform; Boston, MA	https://www.pathai.com/
R version 3.6.0.		https://bioweb.pasteur.fr/packages/pack@R@3.6.0

**Table 1.** Gender-associated distinct patterns of the hepatic and splenic immune responses during aging

Immunological patterns	Male	Female
CD4^+^/CD8^+^ T cells	hepatic CD4^+^ > CD8^+^ splenic CD4^+^ > CD8^+^	hepatic CD4^+^ > CD8^+^ splenic CD4^+^ > CD8^+^
CD4^+^ Th cells	hepatic Th17 > Th1 > Th2 splenic Th17 > Th1 > Th2	hepatic Th1 = Th17 = Th2 splenic Th1 = Th17 > Th2
NKT/NK cells	hepatic NKT > NK splenic NKT = NK	hepatic NKT = NK splenic NKT = NK
Hepatic T cells	CD8^+^ Tcm > Tn > Te = Tem CD4^+^ Tcm > Te = Tem > Tn	CD8^+^ Te = Tem = Tcm = Tn CD4^+^ Te = Tem > Tcm > Tn
Splenic T cells	CD8^+^ Tcm > Tn > Te = Tem CD4^+^ Tcm > Te = Tn > Tem	CD8^+^ Tcm > Tn > Te = Tem CD4^+^ Tcm > Te > Tn > Tem

**Table 2.** Patterns of the hepatic and splenic immune responses associated with the progression or inhibition of HCC

Immunological patterns	Tumor progression	Partial rescue from HCC	Complete rescue from HCC
CD4^+^/CD8^+^ T cells	hepatic CD8^+^ > CD4^+^ splenic CD8^+^ > CD4^+^	hepatic CD8^+^ = CD4^+^ splenic CD8^+^ > CD4^+^	hepatic CD8^+^ > CD4^+^ splenic CD8^+^ = CD4^+^
CD4^+^ Th cells	hepatic Th1 > Th17 > Th2 splenic Th1 = Th17 > Th2	hepatic Th1 = Th17 > Th2 splenic Th1 > Th17 > Th2	hepatic Th1 = Th17 = Th2 splenic Th1 = Th17 > Th2
NKT/NK cells	hepatic NKT > NK splenic NKT > NK	hepatic NKT = NK splenic NKT = NK	hepatic NKT = NK splenic NKT = NK
Macrophages	hepatic M1 > M2 splenic M1 > M2	hepatic M1 > M2 splenic M1 > M2	hepatic M1 = M2 splenic M1 > M2
Hepatic T cells	CD8^+^ and CD4^+^ Te > Tcm	CD8^+^ and CD4^+^ Te > Tcm	CD8^+^ and CD4^+^ Te > Tcm

## Highlights

- A multi-dimensional analysis reveals distinct immunological patterns inhibiting HCC
- Predominant Th1, but not Th17, inflammatory pattern promotes HCC
- An equilibrium Th1 = Th17 = Th2, NKT = NK, M1 = M2 pattern in the liver inhibits HCC
