# Supplementary material for: Distinct hepatic immunological patterns are associated with the progression or inhibition of hepatocellular carcinoma
Source: Cell Rep. Author manuscript; Available in PMC 2022 Apr 22. (PMC9028248; doi:10.1016/j.celrep.2022.110454)
Supplement: 1 [file NIHMS1785222-supplement-1.pdf]

Cell Reports, Volume *38*

Supplemental information

Distinct hepatic immunological patterns are associated with the progression or inhibition of hepatocellular carcinoma

Faridoddin Mirshahi, Hussein F. Aqbi, Madison Isbell, Saeed H. Manjili, Chunqing Guo, Mulugeta Saneshaw, Mikhail Dozmorov, Archit Khosla, Katy Wack, Oscar M. Carrasco-Zevallos, Michael O. Idowu, Xiang-Yang Wang, Arun J. Sanyal, Masoud H. Manjili, and Dipankar Bandyopadhyay

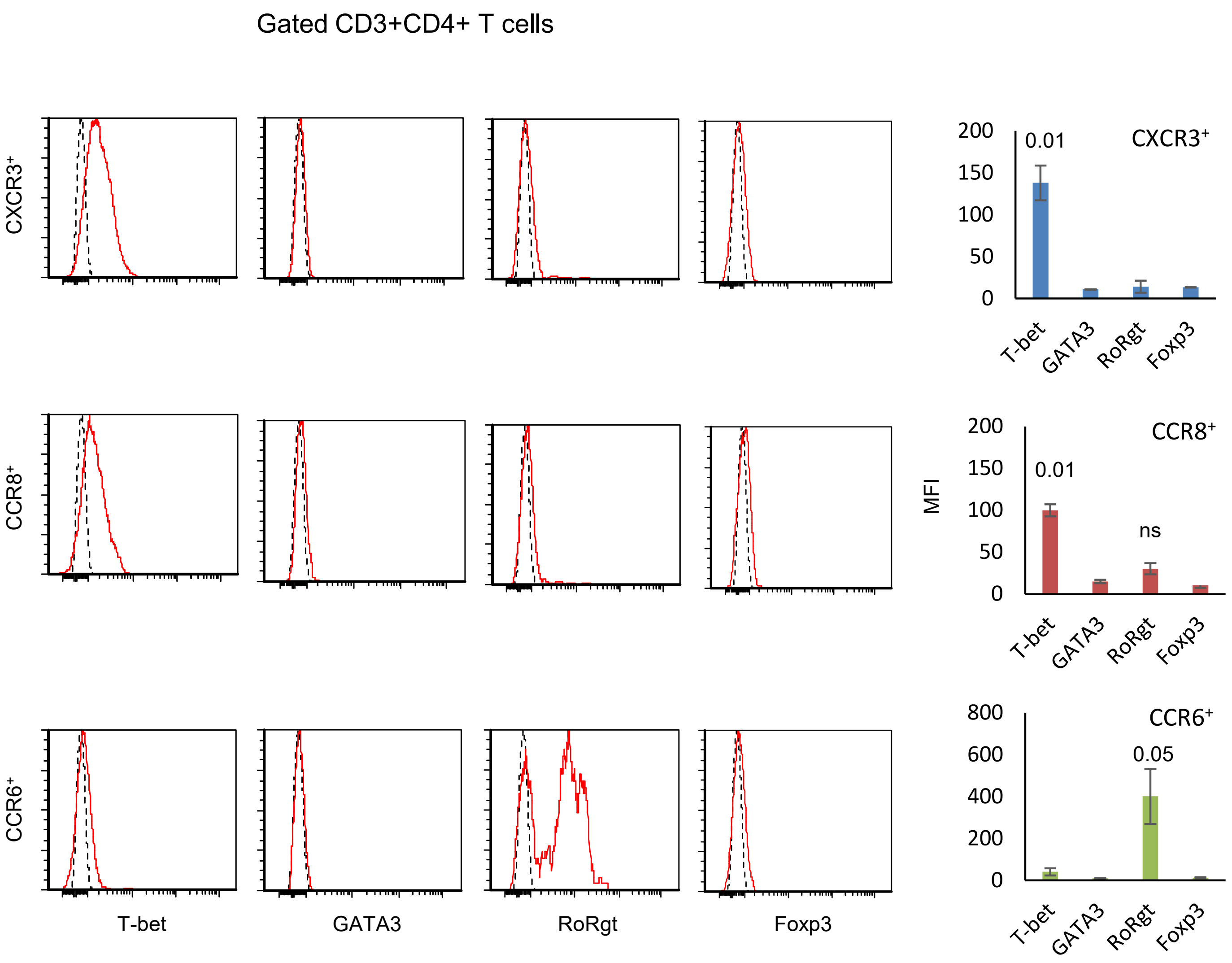

Figure S1. Expression of T-bet, GATA3, RORγ*t* and Foxp3 in CXCR3⁺, CCR8⁺ or CCR6⁺ Th cells. Gated FVS- viable cells in lymphocyte region of the liver of DIAMOND mice being on WD for 24 weeks were further gated on $CD3^{+}CD4^{+}CXCR3^{+}$, $CCR8^{+}$ or $CCR6^{+}$ cells for the expression of transcription factors for Th1 (T-bet), Th2 (GATA3), Th17 (RORγ*t*) or Foxp3 (Tregs). Related to Figures 2-5.

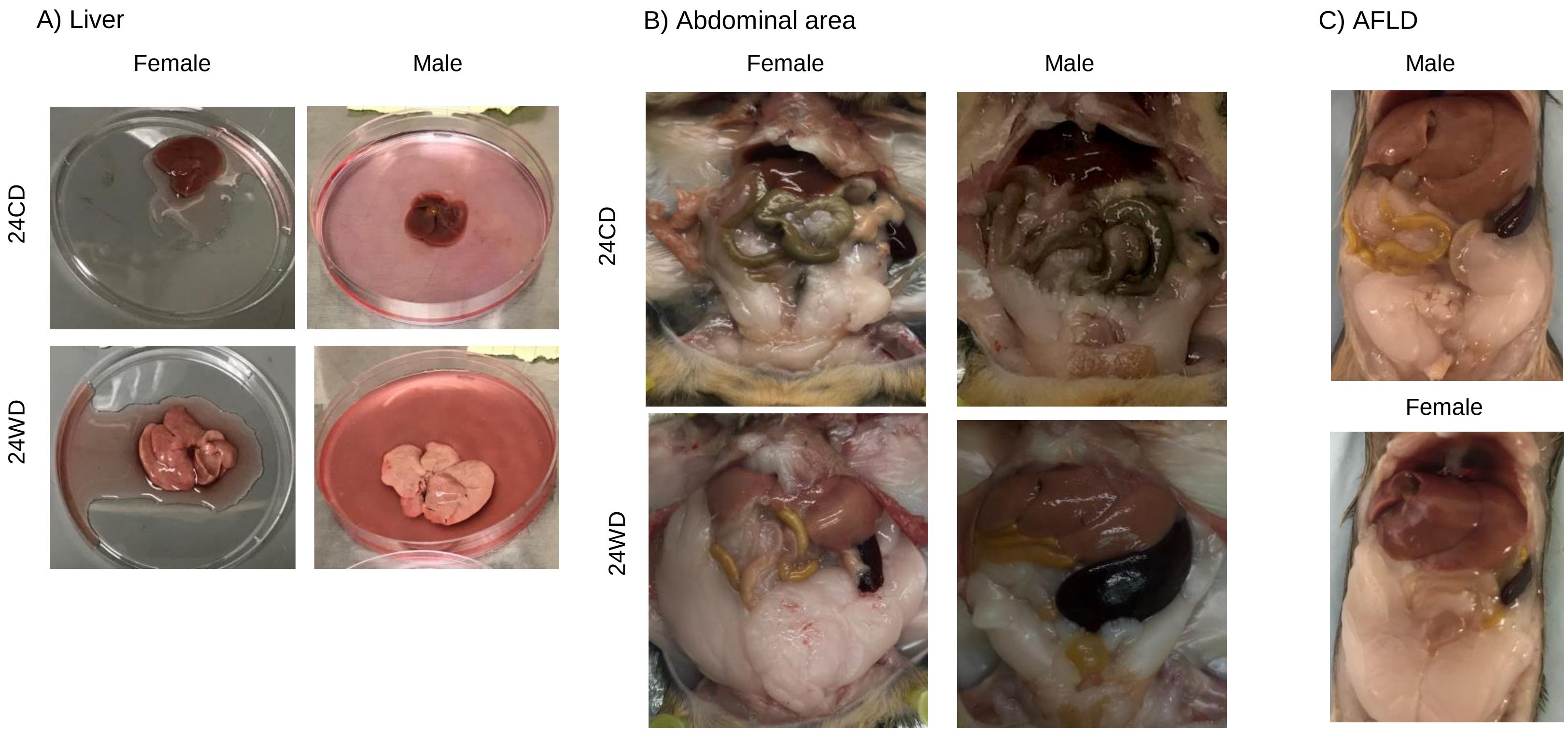

Figure S2. Female and male DIAMOND mice tend to deposit fat predominantly in the abdominal region and in the liver, respectively, during WD. Pictures of liver (A) or abdominal region (B) taken from male or female DIAMOND mice after 24 weeks of being on a CD (24CD) or WD (24WD). Data represents 5 mice per group. C) Alcoholic fatty liver disease (AFLD) in DIAMOND mice after 24 weeks of receiving 40% alcohol in their daily drinking water while being on a WD. Related to Figure 1.

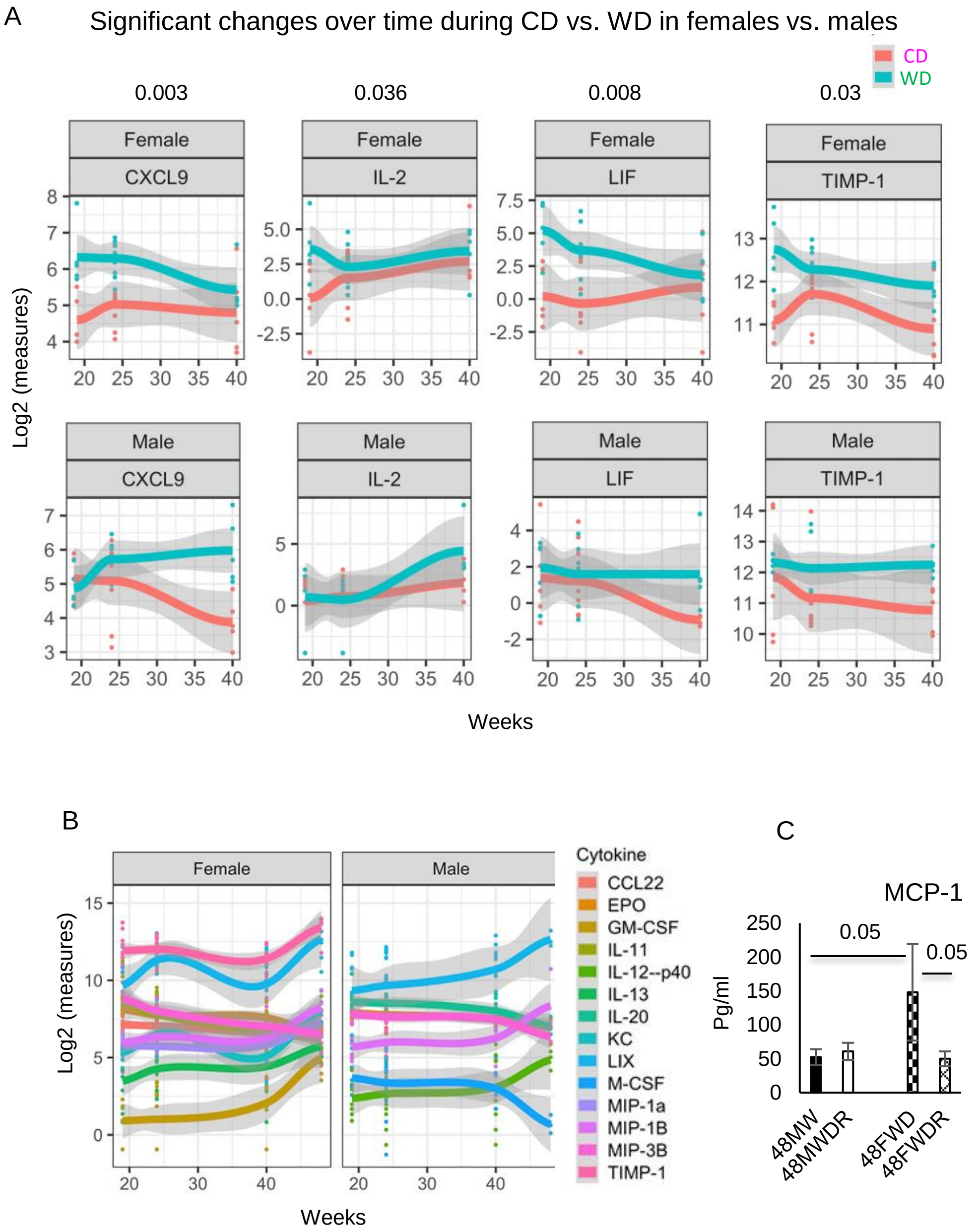

Figure S3. Distinct inflammatory cytokines/chemokines significantly changed in female and male DIAMOND mice during WD. A) Sera were collected from DIAMOND mice (5-9 mice/group) at weeks 19, 24, or 40 of being on a CD or WD, and analyzed for 44 cytokines/chemokines in association with time using multivariate linear regression. The overall time trend, summarized across subjects, is presented for all cytokines/chemokines which have a significant time trend in during CD vs. WD in females vs. males. **B**) The overall time trend, summarized across subjects, is presented only for cytokines/chemokines which have a significant time trend. Results are separated by gender. X-axis - Time in weeks, Y-axis - Log2 of cytokine measure. **C**) Chemokines with a significant change between male mice and female mice after 48 weeks of being on WD (48MWD and 48FWD) or between female mice after 48 weeks of being on WD (48FWD) and female mice after diet reversal at 36 weeks of being on WD and continue on CD for additional 12 weeks (48FWDR). Data represent 4-5 mice/group. Related to Figure 2.

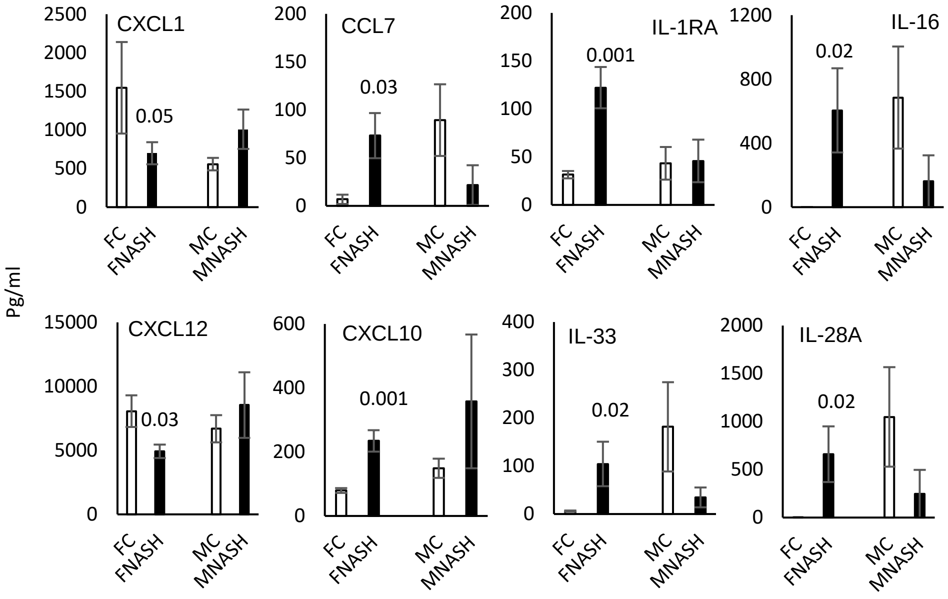

Figure S4. **Distinct patterns of systemic inflammatory cytokines in women or men with NASH.** Sera collected from healthy control females (FC, n=5) or males (MC, n=5) as well as female with NASH (FNASH, n=14) or male with NASH (MNASH, n=4) were subjected to multiplex analysis of 65 human cytokines/chemokines (Eve Technologies, Calgary, Canada). Data represent cytokines/chemokines with significant changes during NASH compare to healthy controls, using Non-Parametic Wilcoxon Exact Test. Related to Figure 2.

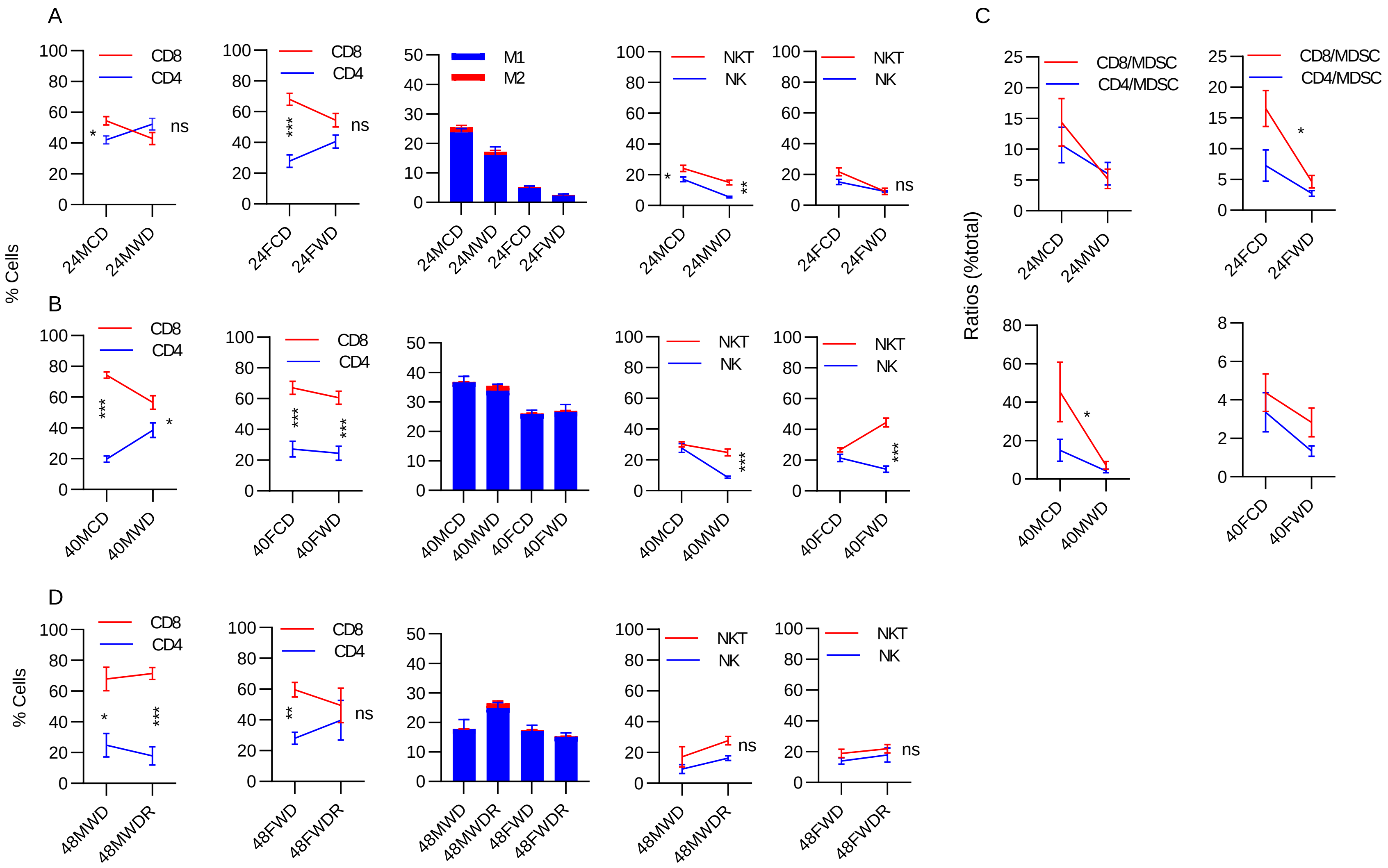

Figure S5. **Splenic immune responses during the progression of and a rescue from HCC**. Female or male DIAMOND mice received CD or WD for 24 (24FCD, 24FWD, 24MCD, 24MWD) and 40 weeks, starting at 10 weeks of age. A group of female or male mice were set aside to receive WD for 48 weeks (48FWD or 48MWD), or being on WD for 36 weeks and received a diet reversal to CD for an additional 12 weeks through week 48 (48MWDR and 48FWDR). **A-B**) FVS- viable splenocytes gated for $CD3^+$ T cells and analyzed for the percentage of $CD8^+$ and $CD4^+$ T cells in males or females being on a CD for WD or 24 weeks (24MCD, 24FCD, 24MWD, 24FWD) or 40 weeks (40MCD, 40FCD, 40MWD, 40FWD). Gated FVS^- viable cells were also analyzed for $F4/80^+CD68^+CD260^-$ M1 cells or $F4/80^+CD68^{+/-}CD260^+$ M2 cells, $CD3^-CD4^-CD8^-CD49b^+$ NK cells or $CD3^+CD4^-CD8^-CD49b^+$ NKT cells. **C**) Percent total cells were analyzed for the ratio of $CD8^+$ T cells to MDSC (CD8/MDSC) or $CD4^+$ T cells to MDSC (CD4/MDSC). D) The splenic T cells, NK cells, NKT cells, M1 and M2 macrophages were also analyzed in males and females after 48 weeks of being on WD (48MWD and 48FWD) as well as those on a diet reversal (48MWDR and 48FWDR). Related to Figure 4.

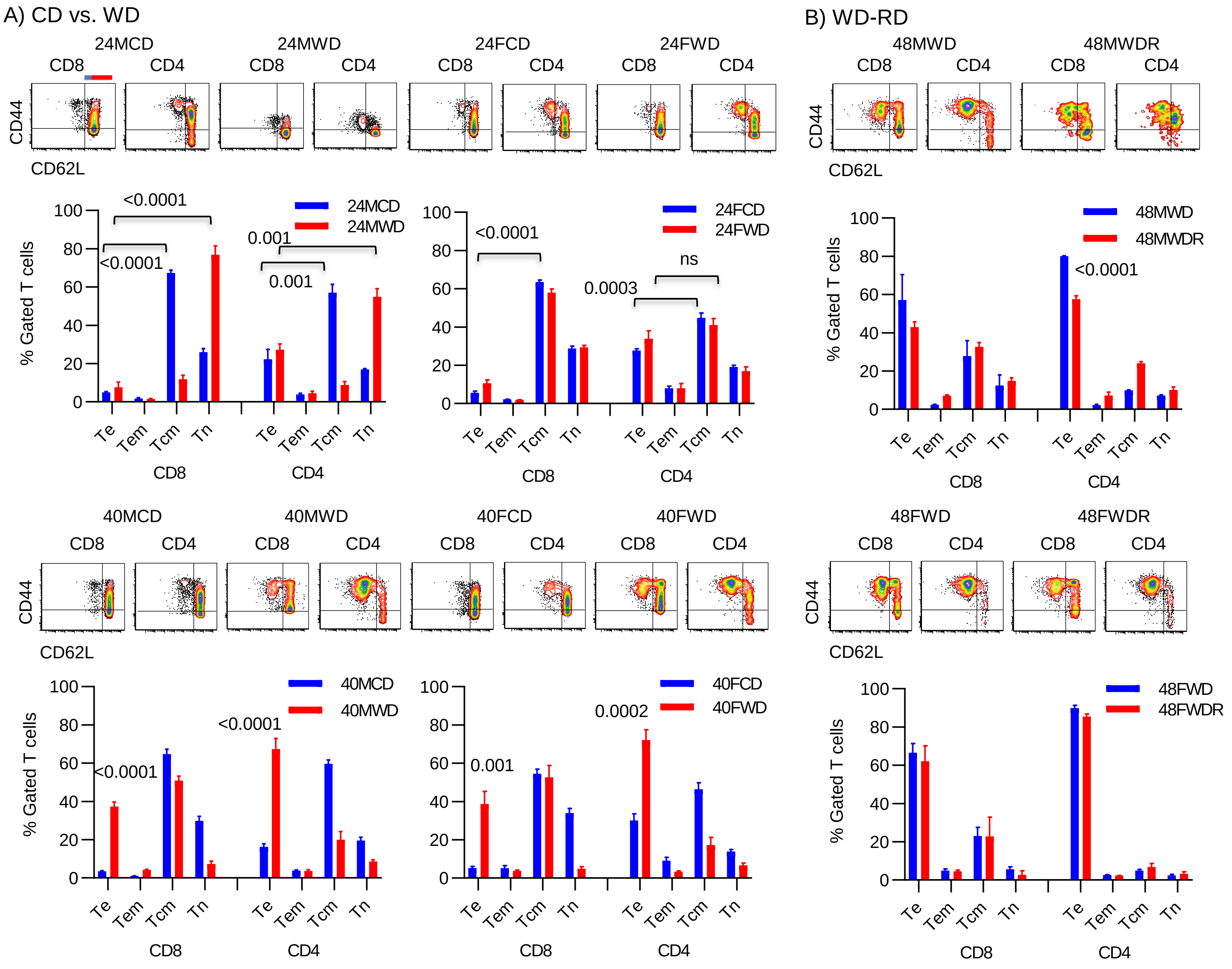

Figure S6. The splenic CD8⁺ and CD4⁺ Te cells become predominant during HCC and a rescue from HCC. Female or male DIAMOND mice were fed CD or WD for 24 weeks (24FCD, 24FWD, 24MCD, 24MWD) and 40 weeks, starting at 10 weeks of age. A group of female or male mice set aside to receive WD for 48 weeks (48FWD or 48MWD), or put back on CD at 36 weeks of being on WD, and continue on CD for an additional 12 weeks through week 48 (48MWDR and 48FWDR). **A-B)** FVS-viable hepatic $CD8^{+}$or $CD4^{+}$ T cells were analyzed for the percentage of Te ($CD44^{+}CD62L$-), Tem (blue line, $CD44^{+}CD62L^{low}$), Tcm (red line, $CD44^{+}CD62L^{high}$) and Tn ($CD44$-$CD62L^{+}$) cells. Related to Figure 5.

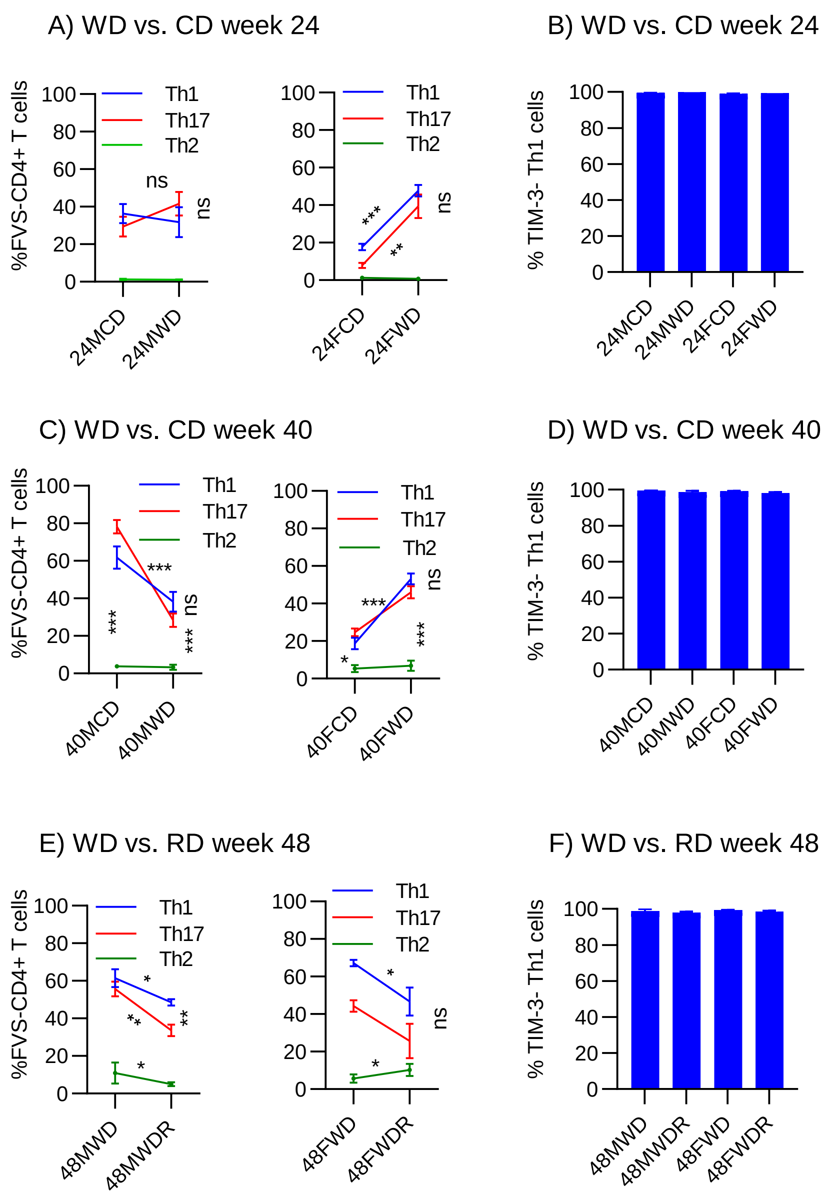

Figure S7. Modulation of the splenic Th1, Th2 and Th17 cells during the progression of or a rescue from HCC. **A**) FVS$^-$ viable splenocytes gated for CD4$^+$ T cells were analyzed for the percentage of Th1 (CD3$^+$CD4$^+$CXCR3$^+$CCR5$^-$), Th2 (CCR8$^+$IL-3Rα^-) or Th17 (CD3$^+$CD4$^+$CCR6$^+$CD161$^-$) cells after 24 weeks of being on a CD or WD. **B**) The splenic Th1 cells were analyzed for the expression of TIM-3. **C**) FVS$^-$ viable splenocytes gated for CD4$^+$ T cells were analyzed for the percentage of Th1, Th2 or Th17 cells after 40 weeks of being on a CD or WD. **D**) The splenic Th1 cells were analyzed for the expression of TIM-3. **E**) FVS$^-$ viable splenocytes gated for CD4$^+$ T cells were analyzed for the percentage of Th1, Th2 or Th17 cells after 48 weeks of being on a WD or during RD. **F**) The splenic Th1 cells were analyzed for the expression of TIM-3, after 48 weeks of being on a WD or during a RD. Related to Figure 5.
